# Prenatal environmental exposures associated with sex differences in childhood obesity and neurodevelopment

**DOI:** 10.1186/s12916-023-02815-9

**Published:** 2023-04-12

**Authors:** Alejandro Cáceres, Natàlia Carreras-Gallo, Sandra Andrusaityte, Mariona Bustamante, Ángel Carracedo, Leda Chatzi, Varun B. Dwaraka, Regina Grazuleviciene, Kristine Bjerve Gutzkow, Johanna Lepeule, Léa Maitre, Tavis L. Mendez, Mark Nieuwenhuijsen, Remy Slama, Ryan Smith, Nikos Stratakis, Cathrine Thomsen, Jose Urquiza, Hannah Went, John Wright, Tiffany Yang, Maribel Casas, Martine Vrijheid, Juan R. González

**Affiliations:** 1 Instituto de Salud Global de Barcelona (ISGlobal), 08003 Barcelona, Spain; 2 Centro de Investigación Biomédica en Red en Epidemiología Y Salud Pública (CIBERESP), Madrid, Spain; 3 Department of Mathematics, Escola d’Enginyeria de Barcelona Est (EEBE), Universitat Politècnica de Catalunya, 08019 Barcelona, Spain; 4 Department of Environmental Science, Vytautas Magnus University, 44248 Kaunas, Lithuania; 5 Department of Health and Experimental Sciences, Universitat Pompeu Fabra (UPF), Barcelona, Spain; 6 Center for Genomic Regulation (CRG), Barcelona, Institute of Science and Technology (BIST), Barcelona, Spain; 7 Medicine Genomics Group, Centro de Investigación Biomédica en Red Enfermedades Raras (CIBERER), CIMUS, University of Santiago de Compostela, Santiago de Compostela, Spain; 8 Galician Foundation of Genomic Medicine, Instituto de Investigación Sanitaria de Santiago de Compostela (IDIS), Servicio Gallego de Salud (SERGAS), Galicia, Santiago de Compostela Spain; 9 Department of Preventive Medicine, Keck School of Medicine, University of Southern California, Los Angeles, USA; 10 TruDiagnostic, Lexington, KY USA; 11 Division of Climate and Environmental Health, Norwegian Institute of Public Health, 0456 Oslo, Norway; 12 Institut National de La Santé Et de La Recherche Médicale (Inserm) and Université Grenoble-Alpes, Institute for Advanced Biosciences (IAB), Team of Environmental Epidemiology Applied to Reproduction and Respiratory Health, Grenoble, France; 13 Bradford Institute for Health Research, Bradford Teaching Hospitals NHS Foundation Trust, Bradford, UK; 14 Department of Mathematics, Universitat Autònoma de Barcelona, Bellaterra, 08193 Barcelona , Spain

**Keywords:** Prenatal environment, Sexual dimorphism, Childhood obesity, Neurodevelopment, DNA methylation, Causal inference, Multiexposure profile

## Abstract

**Background:**

Obesity and neurodevelopmental delay are complex traits that often co-occur and differ between boys and girls. Prenatal exposures are believed to influence children’s obesity, but it is unknown whether exposures of pregnant mothers can confer a different risk of obesity between sexes, and whether they can affect neurodevelopment.

**Methods:**

We analyzed data from 1044 children from the HELIX project, comprising 93 exposures during pregnancy, and clinical, neuropsychological, and methylation data during childhood (5–11 years). Using exposome-wide interaction analyses, we identified prenatal exposures with the highest sexual dimorphism in obesity risk, which were used to create a multiexposure profile. We applied causal random forest to classify individuals into two environments: E1 and E0. E1 consists of a combination of exposure levels where girls have significantly less risk of obesity than boys, as compared to E0, which consists of the remaining combination of exposure levels. We investigated whether the association between sex and neurodevelopmental delay also differed between E0 and E1. We used methylation data to perform an epigenome-wide association study between the environments to see the effect of belonging to E1 or E0 at the molecular level.

**Results:**

We observed that E1 was defined by the combination of low dairy consumption, non-smokers’ cotinine levels in blood, low facility richness, and the presence of green spaces during pregnancy (OR_interaction_ = 0.070, *P* = 2.59 × 10^−5^). E1 was also associated with a lower risk of neurodevelopmental delay in girls, based on neuropsychological tests of non-verbal intelligence (OR_interaction_ = 0.42, *P* = 0.047) and working memory (OR_interaction_ = 0.31, *P* = 0.02). In line with this, several neurodevelopmental functions were enriched in significant differentially methylated probes between E1 and E0.

**Conclusions:**

The risk of obesity can be different for boys and girls in certain prenatal environments. We identified an environment combining four exposure levels that protect girls from obesity and neurodevelopment delay. The combination of single exposures into multiexposure profiles using causal inference can help determine populations at risk.

**Supplementary Information:**

The online version contains supplementary material available at 10.1186/s12916-023-02815-9.

## Background

Boys and girls develop differently. For instance, their immune response to infections differs from an early age, their brains grow at different rates, and the prevalence of numerous common diseases, like obesity, is also different [1–3]. As reported by Shah et al., 65% of the countries around the world and 96% of high-income countries reported a greater prevalence of obesity for boys than girls in children aged 5–9 years old [3, 4]. Given the contrasting paths of development, it is remarkable that biomedical studies typically consider sex as a confounder rather than the main effect or an effect modifier [5]. Exposome studies, in particular, are characterized by the acquisition of massive amounts of data at individual and population levels [6]. A crucial goal of these studies is to inform the likely conditions for which a given public health intervention would be optimal, such that the best intervention is applied at the right time to the right population [7]. However, as the main difference between individuals is sex, exposome studies aiming at improving precision medicine and precision public health cannot do without considering how environmental risk factors affect sexual dimorphism in development and disease.

From a mechanistic context, studying the factors that increase sexual dimorphic outcomes of disease can offer important insights into its etiology and comorbidities, and inform of possible interventions and targeted treatments. Important advancements have been made in studying sex-related risk factors for diseases like cancer, Alzheimer’s, and autoimmune diseases [8]. However, a relevant component of these age-related diseases is hormonal regulation. Studying sex differences in preteens offers not only the opportunity for identifying targeted treatments for early-age illnesses but also to explore disease mechanisms unlikely influenced by sex hormones that may also onset early in life. Previous research has, for instance, underlined that maternal factors during pregnancy can affect disease outcomes later in life [9] and, therefore, motivates the question of which pregnancy factors may promote later sexual dimorphism in disease.

Environmental exposures likely orchestrate environments that are more toxic to one sex than to the other one. However, methods to determine such multiple-exposure environments are not readily available. We have developed a method of causal modeling, based on causal random forest, that can determine profiles of multiple exposures that are associated with high sexual dimorphism [10]. Here, we aimed to adapt our method to determine which combination of prenatal exposures can produce an environment where girls are more protected from obesity than boys during the preteen years. Furthermore, obesity in children is associated with lower cognitive function, particularly inhibitory control and working memory, critical for academic achievement [11]. Obesity often co-occurs with neurodevelopmental disorders, particularly in boys [12]. Therefore, we also evaluated whether the environment of high sexual dimorphism in obesity also shows a significant sexual dimorphism in non-verbal intelligence, working memory, attention, and ADHD.

Finally, we investigated whether the protective environment may be associated with epigenetic changes since many exposures during pregnancy are associated with specific methylation profiles [13]. This analysis may provide information about the molecular pathways that may be participating in the association between the environment and the sexual differences in obesity and neurodevelopment.

Here, we aimed to (1) combine multiple exposure levels to define an environment with high sexual dimorphism in obesity risk; (2) given the correlation between obesity and neurodevelopmental delay in children, we also enquired if the subpopulation exposed to this environment shows a significant sexual dimorphism in neurodevelopment; and (3) we then hypothesized that the individuals who belong to such an environment can be characterized by specific patterns of DNA methylation.

## Methods

### Study population

We analyzed data from The Human Early Life Exposome (HELIX). This is a multi-center study that included a total of 1301 mother–child pairs from six existing birth cohorts in Europe: BIB (Born in Bradford; the UK) [14], EDEN (Etude des Déterminants pré et postnatals du développement et de la santé de l’Enfant; France) [15], INMA-SAB (Infancia y Medio Ambiente; Spain; subcohort Sabadell) [16], KANC (Kaunas cohort; Lithuania) [17], MoBa (The Norwegian Mother, Father and Child Cohort study; Norway) [18]), and Rhea (Greece) [19]. The pairs participated in a common, completely harmonized, follow-up examination, when children were between 5–11 years old to fully characterize the pregnancy and childhood exposome [20]. During the clinical examination, urine (pooled spot urine samples from before bedtime and first morning void) and blood samples were collected from the children. Urine and blood samples previously collected from mothers during pregnancy were also available for biomarkers of chemical exposure assessment. In our analyses, we selected the individuals who had data on prenatal exposures, performed the clinical and neurodevelopment examination, and had methylation data (*N* = 1044). All studies received approval from the ethics committees of the centers involved and written informed consent was obtained from all participants. Cohort characteristics are shown in Table 1.

**Table 1:** Characteristics of HELIX cohort. Clinical characteristics of children during pregnancy and follow-up

**Children assessed at follow-up**	***N* = 1044**
**Sex, male**	571 (54.6%)
**Cohort**	
BIB (UK)	90 (8.6%)
EDEN (France)	135 (12.9%)
INMA (Spain)	198 (19.0%)
KANK (Lithuania)	196 (18.8%)
MOBA (Norway)	239 (22.9%)
RHEA (Greece)	136 (17.8%)
**Age in years, mean (range)**	7.9 (5.4–11.9)
**BMI (kg/m**^**2**^**), median (range)**	16.3 (12.2–29.5)
Obesity	62 (5.9%) – F: 23 (4.9%) – M: 39 (6.8%)
**Raven’s matrices, median (range)**	27 (9–36)
Affected	189 (18.2%) – F: 78 (16.6%) – M: 111 (19.5%)
**N-back (2-back accuracy), median (range)**	0.91 (0.36–1)
Affected	104 (12.9%) – F: 46 (12.6%) – M: 58 (13.0%)
**ANT (accuracy), median (range)**	0.97 (0.51–1)
Affected	206 (20.0%) – F: 72 (15.6%) – M: 134 (23.7%)
**ADHD**	104 (10.0%) – F: 27 (5.8%) – M: 77 (13.6%)
**Prenatal characteristics**	***N* = 1044**
**Mother’s age during pregnancy, mean (range)**	30.9 (16–34)
**Mother’s BMI during pregnancy, median (range)**	23.6 (15.8–51.4)
**Mother’s weight gain during pregnancy, mean (range)**	13.9 (0–40)
**Maternal education**	
Primary school	119 (11.3%)
Secondary school	359 (34.3%)
University degree or higher	566 (54.2%)
**Gestational age, median (range)**	40 (30.8–44.1)
**Year of birth, median (range)**	2006 (2003–2009)
**Number of parents native from the country cohort, mean (range)**	1.9 (02)
Parity	0.69 (0–2)

*BMI* body mass index, *ANT* attention network test, *ADHD* attention deficit hyperactive disorder, *F* female, *M* male

### Clinical outcomes

Height and weight measurements were measured during the clinical visit performed at ages 5 to 11 years. These measurements were converted to body mass index (BMI in kg/m^2^) for age-and-sex *z*-scores using the international WHO reference curves to allow comparison with other studies [21]. Obese children were defined as those above the age- and sex-specific 95th percentile, as recommended by WHO.

**Fig. 1:**
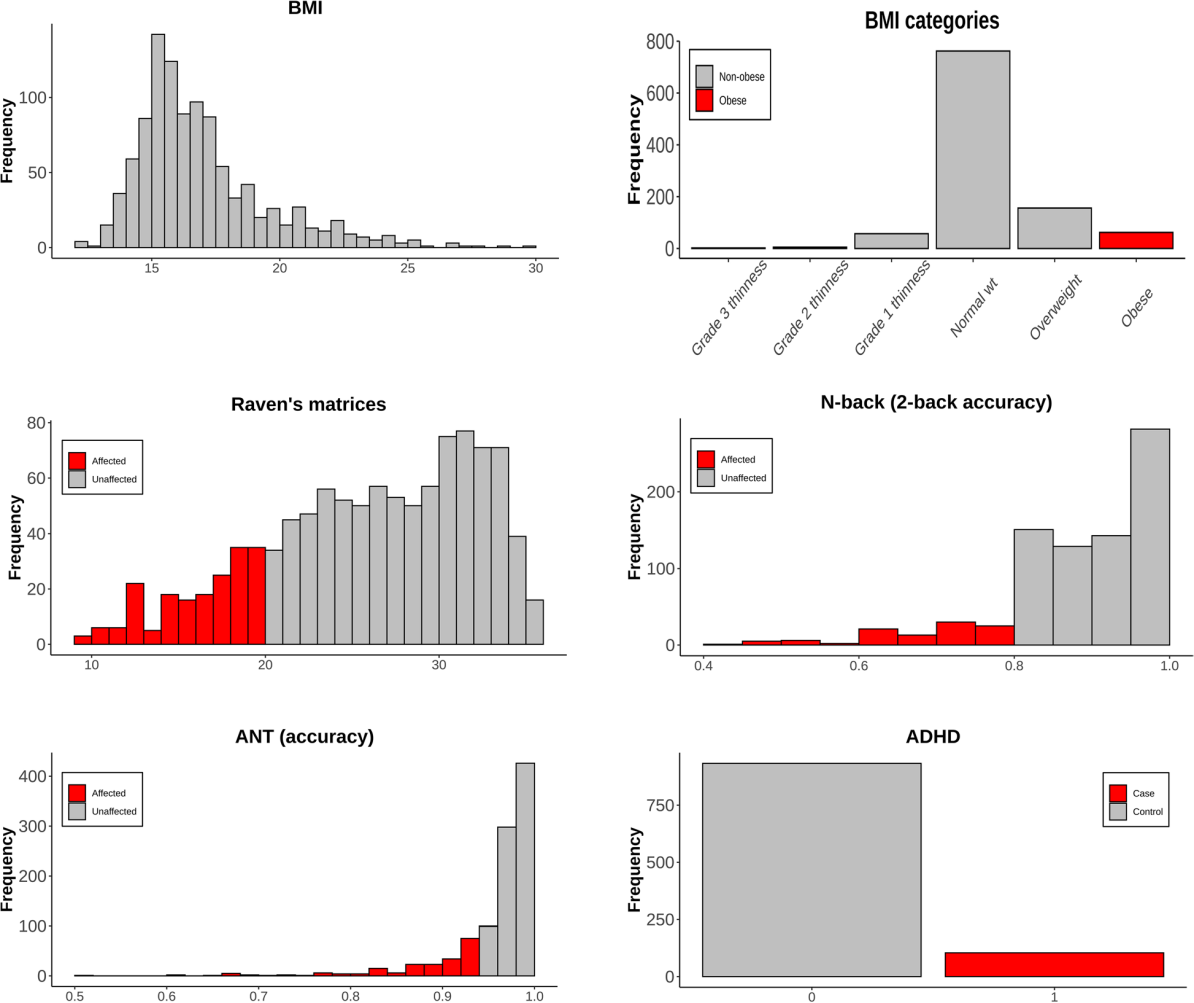
Distributions of clinical outcomes in the HELIX study. Analyses were performed for categorized variables shown in gray (reference) and red (affected)

Neurodevelopmental outcomes were assessed through a battery of internationally standardized, non-linguistic, and culturally blind computer tests also at ages 5 to 11 years. We assessed working memory, attention, and general non-verbal intelligence with the N-back test [22], the attention network test (ANT) [23], and Raven’s colored progressive matrices [24]; respectively. The tests were administered in a standardized way by trained field workers through study-provided laptops. The outcomes did not distribute normally. We dichotomized them, taking as cases individuals with outcomes below the first quintiles (20%), which clearly captured the long lower tail of the outcomes’ distributions (Fig. 1). These percentiles were chosen because they allowed the selection of the lowest performers in the outcomes using a single criterion and preserving a representative number of individuals within the groups. We thus studied as clinical outcomes the events of having these cognitive abilities affected. We also considered ADHD diagnosis.

We considered as common covariates (covariates used in all the analyses) 10 variables, based on Maitre et al. [25]. These covariates are cohort, year of birth, mother’s BMI, mother’s weight gain during pregnancy, gestational age, mother’s age during pregnancy, mother’s education, whether parents were native from the country cohort, parity, and children’s age at clinical assessment (Table 1).

### Pregnancy exposome

HELIX has collected a wide range of exposures measured during two main windows: a prenatal window including the pregnancy period and a postnatal window including the exposome data of children at the same time as omics sampling (childhood). In this study, we only considered the first window (pregnancy exposome) which consists of 93 exposures distributed across 17 exposure families, including the urban environment, the chemical exposome, and social and lifestyle factors.

The urban environment includes exposure estimates for built environment, surrounding green and blue spaces, ultraviolet (UV) radiation, road traffic noise levels, air pollution, noise, meteorology, and socioeconomic deprivation index [20, 26]. These exposures were assessed during pregnancy by environmental geographic information systems (GIS) according to their residential addresses. Tobacco smoke and diet were evaluated by questionnaires. Biomarkers of contaminant exposure, like cotinine levels, were measured in appropriate biological samples (urine or blood) collected from mothers during pregnancy. Details on the exposure assessment methods and exposure factors can be seen in the Additional file 1: Supplementary Methods [20, 27–33].

Missing values for all exposures were imputed using the method of chained equations using the *mice* package in R [32], as described in detail elsewhere [33]. When possible, multiple imputation procedure was applied (missing values are imputed stochastically several times). For the imputation process, continuous variables should have a normal distribution. Thus, skewed exposure variables were transformed to achieve normality or categorized if no transformation worked. Exposure variables with their corresponding transformation are described in Additional file 2: Table S1. Exposures with more than 70% of missing values in each cohort were excluded from the imputation process. Therefore, missing values ranged from 1.5% in traffic density to 65% in fast-food intake during pregnancy. Although none of the participants had complete data on all exposures, 95% of individuals had missing values in less than 30% of exposures.

### DNA methylation

One of the main goals of HELIX was to associate multiple environmental factors with omics biomarkers and child health outcomes. For these same children, multi-omics molecular phenotyping was performed, which included measurement of blood DNA methylation (450 K, Illumina), among others.

The DNA was obtained from buffy coat collected in EDTA tubes at 5–11 years of age. Briefly, DNA was extracted using the Chemagen kit (Perkin Elmer) in batches of 12 samples. Samples were extracted by cohort and following their position in the original boxes. DNA concentration was determined in a NanoDrop 1000 UV–Vis Spectrophotometer (ThermoScientific) and with Quant-iT™ PicoGreen® dsDNA Assay Kit (Life Technologies). DNA methylation was assessed using the Infinium Human Methylation 450 beadchip (Illumina), following the manufacturer’s protocol. Preprocessing of methylation data has been described elsewhere [34]. After sample and probe quality control measures, the number of CpG probes analyzed was 371,533, initially available for 1192 subjects. We used the Combat algorithm to remove the batch effects supported by the slide. Methylation levels were expressed as beta values corrected by surrogate variables and CpG sites were annotated to genes by Illumina HM450 manifest file (version 1.2). We discarded the subjects without exposome data and without European ancestry based on genomic data, resulting in 993 individuals for the methylome analysis. We computed blood cell type proportions following Houseman et al. algorithm [35] and Reinius reference panel [36].

### Statistical methods

**Fig. 2:**
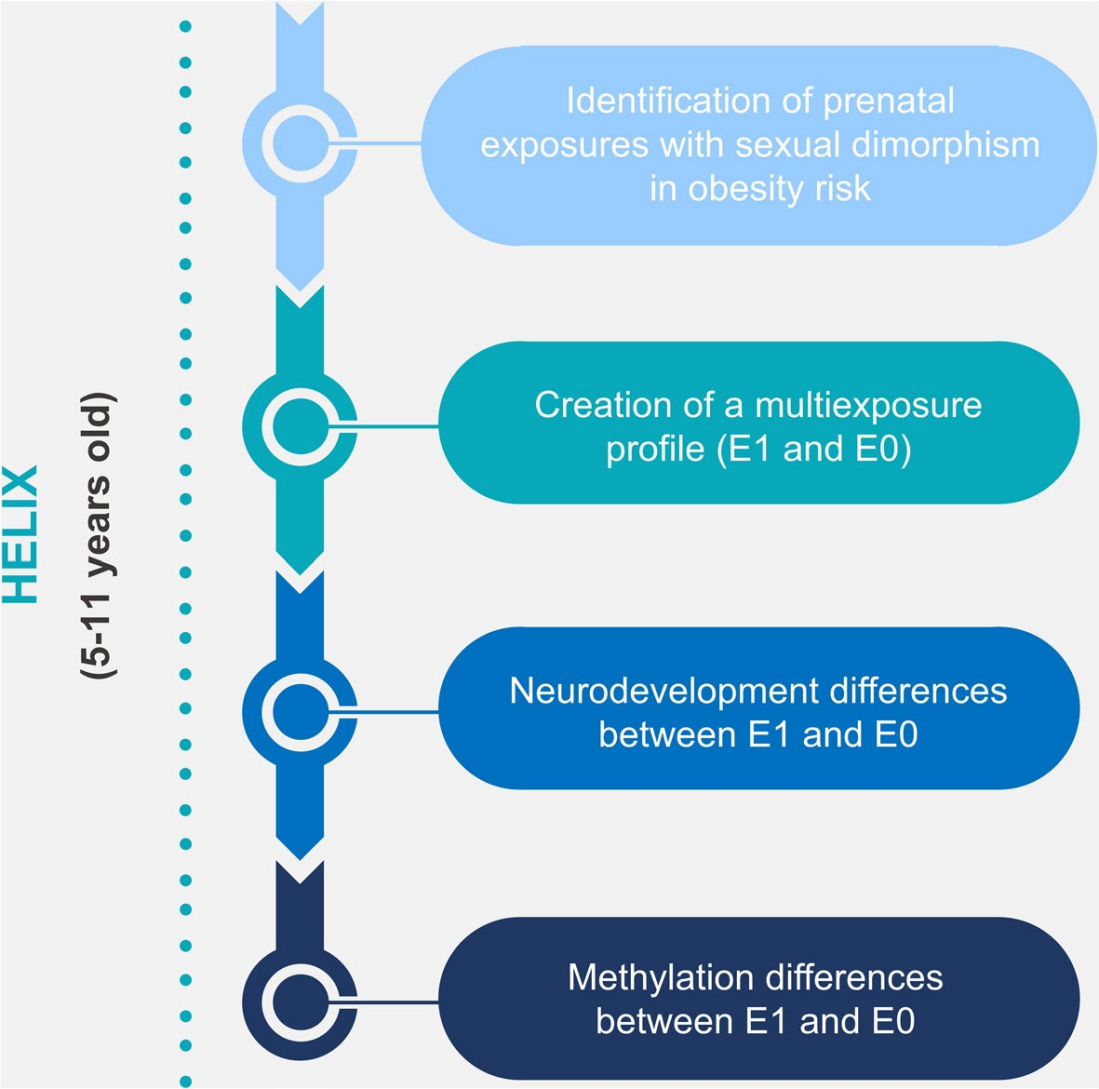
Statistical workflow. The figure shows all the statistical analyses carried out along the paper

Figure 2 shows the statistical workflow.

### Identification of prenatal exposures with sexual dimorphism in obesity risk

We used exposome-wide interaction analyses to determine the exposures whose association with obesity was significantly different between sexes. We assessed the associations between obesity (cases and controls) and the interactions between sex (*S*) and each of the prenatal exposures (*D*_*i*_) using the logistic regression model.

$$E(Y)=\mathrm{logit}^{-1}\left(\alpha_i+\beta_i\left(S\times D_i\right)+{\textstyle\sum_{r=1\dots k\mathit\;}}\gamma_{ir}C_{ri}\right)$$

where *Y* is the obesity status of an individual with sex *S* and *i*th exposure *D*_*i*_. *γ*_*ir*_ are the regression coefficients of the *k* covariates *C*_*ri*_ that included sex, exposure *I*, and the 10 common covariates mentioned before (cohort, year of birth, mother’s BMI, mother’s weight gain during pregnancy, gestational age, mother’s age during pregnancy, mother’s education, whether parents were native from the country cohort, parity, and children age at clinical assessment). *β*_*i*_ were the effects of interest that measure the association between obesity and the interaction between sex and each exposure *i*. We adjusted *p*-values using false discovery rate to correct for multiple comparisons.

### Creation of a multiexposure profile (E1 and E0)

We calculated the residuals of the exposures with nominal significant interactions adjusted by the 10 common covariates. Then, we used these residuals as covariates in causal inference modeling, using causal random forest and taking sex as the treatment variable, to determine which children in HELIX had been in personal environments with significant sexual dimorphism in obesity (female > male or female < male). We then aimed to determine whether the personal environments of the children with one of the significant dimorphisms (F > M or F < M) could be averaged into two prenatal environments, one whose female protection against obesity was stronger than those observed for the individual exposures and the other the opposite. We did not find enough children with negative dimorphism (F > M). We thus created an average environment with highly significant female protection against obesity, which hereinafter we will refer to it as E1. E1 was defined as a binary vector, with one entry for each level of the exposures, indicating whether a given exposure averaged across the children with positive dimorphism was higher or lower than the average across all other children in the training set. We used the multiexposure profile to classify all the individuals in the entire HELIX cohort depending on whether they belong to the E1 or not (E0: F < M and F = M). To this end, we used soft targeting that tested whether they matched the environment in at least 60% of the exposures. The causal inference and the classification into the multiexposure profile associated with the E1 environment were performed with the algorithm *teff*, taking sex as the treatment variable [10] (https://teff-package.github.io/).

### Neurodevelopment differences between E1 and E0

We used the classification of individuals into E1 and E0 to assess their relationship with sex differences in neurodevelopment. For this analysis, we used logistic regression models on the clinical outcomes (working memory, attention and general non-verbal intelligence with the N-back test, ANT, Raven’s colored progressive matrices, and ADHD) and we tested the interaction between the environment (E1/E0) with sex. We adjusted the model by sex, the environment, and the 10 common covariates.

### Methylation differences between E1 and E0

We performed an epigenome-wide association study (EWAS) in the HELIX cohort between E1 and E0. As previously, we used logistic regression models to identify the probes that were differentially methylated between environments. We adjusted the analysis by the 10 common covariates used in previous analyses and counts of different immune cells in the blood. Associations were corrected for multiple comparisons using false discovery rate, as computed by *limma*. For the enrichment analysis, we used *clusterProfiler* Bioconductor package (V.3156). The commented analysis code is available in Additional file 3: Supplementary Code.

## Results

### Sexual dimorphism of clinical outcomes

**Fig. 3:**
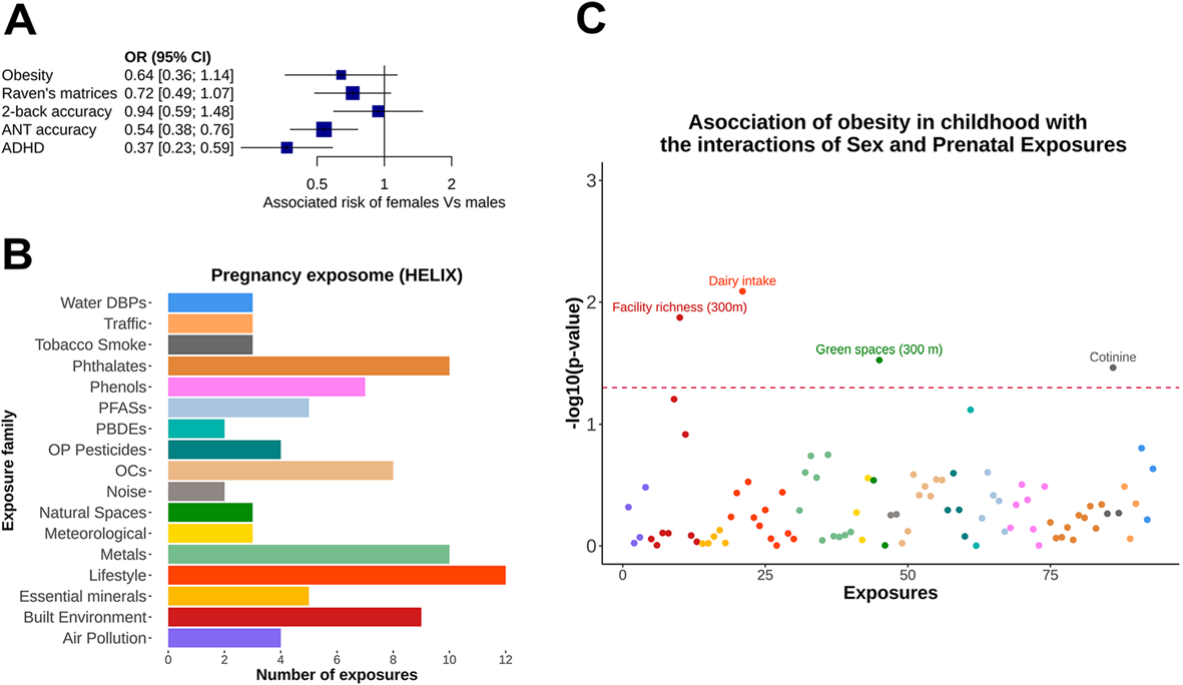
**A** Association of sex with the clinical outcomes, adjusting by covariates.** B** Number of prenatal exposures in each family measured in HELIX. **C** Exposome-wide Manhattan plot. Association of obesity with 93 sex-prenatal exposure interactions (the color follows the exposure family from panel **A**). The dotted line marks nominal significance (*P* = 0.05)

We first assessed whether obesity and the categorized neuropsychological measures were associated with differences between sexes (Fig. 1). We fitted logistic regression models adjusting by the 10 common covariates. Girls showed a lower frequency of obesity than boys, but it was not statistically significant (OR = 0.64, *P* = 0.13, see Fig. 3A). For the neuropsychological measures, we observed that ADHD was lower in girls than boys, consistent with girls’ higher protection in attention difficulty. Both associations were statistically significant (OR = 0.37, *P* = 2.87 × 10^−5^, OR = 0.54, *P* = 4.32 × 10^−4^). For Raven’s matrices and N-back, we did not see significant associations with sex (OR = 0.72, *P* = 0.10, OR = 0.94, *P* = 0.78, respectively) (Fig. 3A).

### Exposome-wide analysis of sex-exposure interactions on obesity

**Fig. 4:**
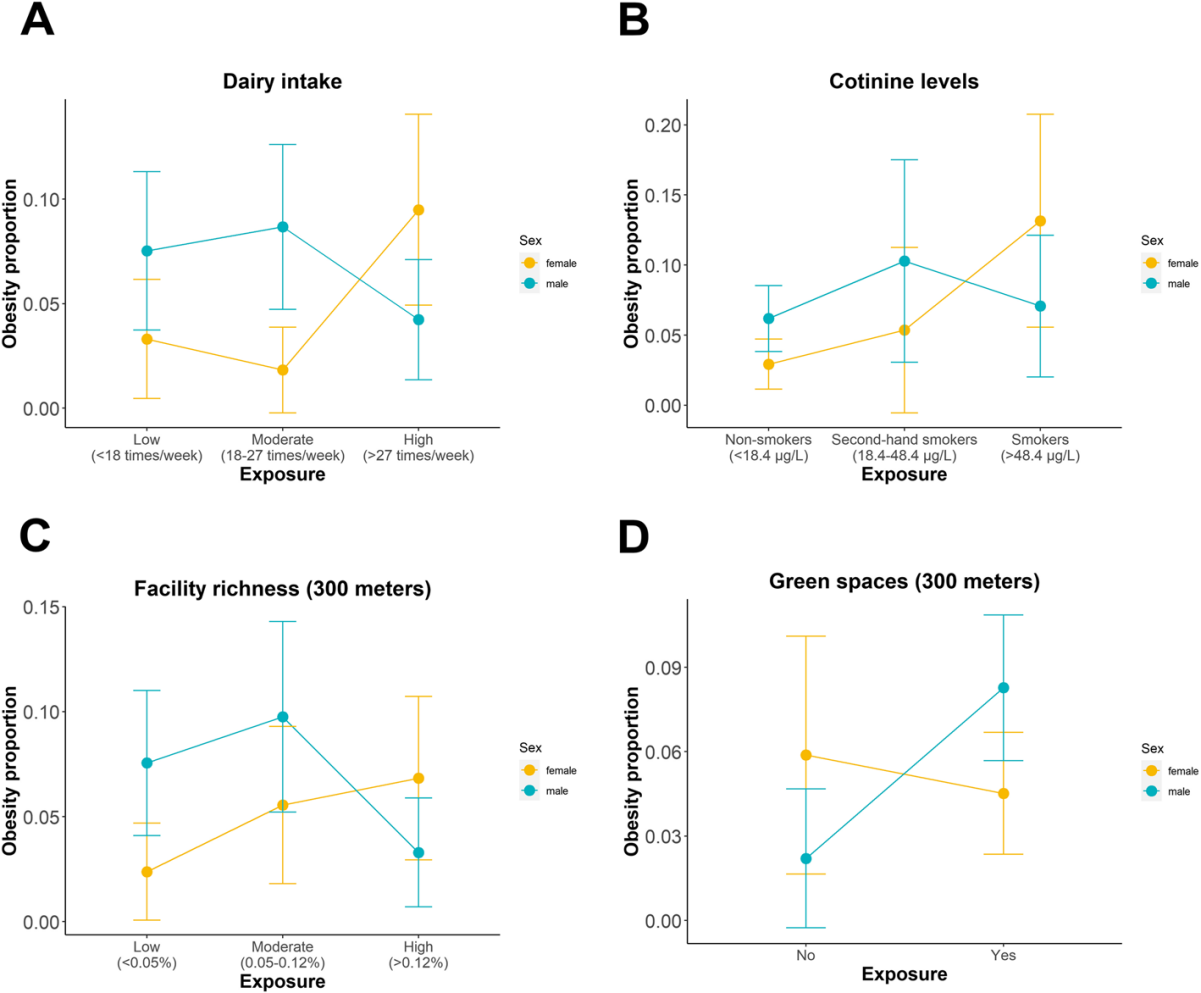
Sex-exposure interaction plots on obesity. **A** Mother’s dairy consumption during pregnancy. The figure shows the highest proportion of obesity in girls with the highest level of dairy consumption. **B** Mother’s cotinine levels in the blood. The highest levels of obesity were observed in girls with high cotinine levels. **C** Facility richness in living neighborhoods of pregnant mothers. A high abundance of facility richness is correlated with a low prevalence of obesity in boys. **D** Green spaces at 300 m from pregnant mothers’ homes. The highest prevalence of obesity was observed in boys with mothers living in the presence of green spaces. The bars represent the 95% confidence intervals for the estimated proportion of obesity

We searched for prenatal exposures that could modulate the association between sex and obesity in childhood. Particularly, we searched for maternal exposure levels in which one sex would be more obese than the other at 5–11 years of age. We performed logistic regressions on obesity for all 93 sex-prenatal exposures interactions, adjusting by the common covariates, sex, and each exposure (Fig. 3B, C). We did not observe any interaction that passed multiple comparison corrections. However, at the nominal level (*P* < 0.05), we observed four interactions between sex (males as reference) and prenatal exposures. First, dairy consumption (OR_intreraction_ = 2.44, *P* = 0.008) is defined as mother’s dairy consumption during pregnancy times per week and categorized as less than 18 times per week (low), between 18 and 27 (moderate), and more than 27 (high). Second, cotinine levels in mothers during pregnancy (OR_intreraction_ = 1.92, *P* = 0.034) are classified into three categories: non-smokers (less than 18.4 µg/L), second-hand smokers (between 18.4 and 48.4 µg/L), and smokers (more than 48.4 µg/L). Third, facility richness (OR_intreraction_ = 1.11, *P* = 0.013) is defined as the percentage of different facility types present compared to the maximum potential number of facility types at a 300-m buffer during the pregnancy period. We categorized this variable into low (less than 0.05%), moderate (between 0.05 and 0,12%), and high abundance (more than 0.12%). Fourth, the presence of green spaces (OR_intreraction_ = 0.27, *P* = 0.029), answering the question of whether the mother lived within a distance of 300 m of green space during the pregnancy period (yes/no). A stratified analysis by sex of the association between obesity and the significant exposures revealed that dairy consumption and cotinine levels were risk factors only for girls (OR = 2.88, *P* = 0.0009; OR = 1.91, *P* = 0.0128) while facility richness and green spaces were protective and risk factors for boys, respectively (OR = 0.92, *P* = 0.005; OR = 5.06, *P* = 0.007), see Fig. 4. We finally asked the extent to which the four exposures were correlated between each other. Interestingly, we found weak but significant Pearson’s correlations of facility richness with dairy intake (r =  − 0.11, *P* = 0.0002) and cotinine levels (r = 0.07, *P* = 0.01).

### Exposure environment of high differences in obesity risk between sexes

**Fig. 5:**
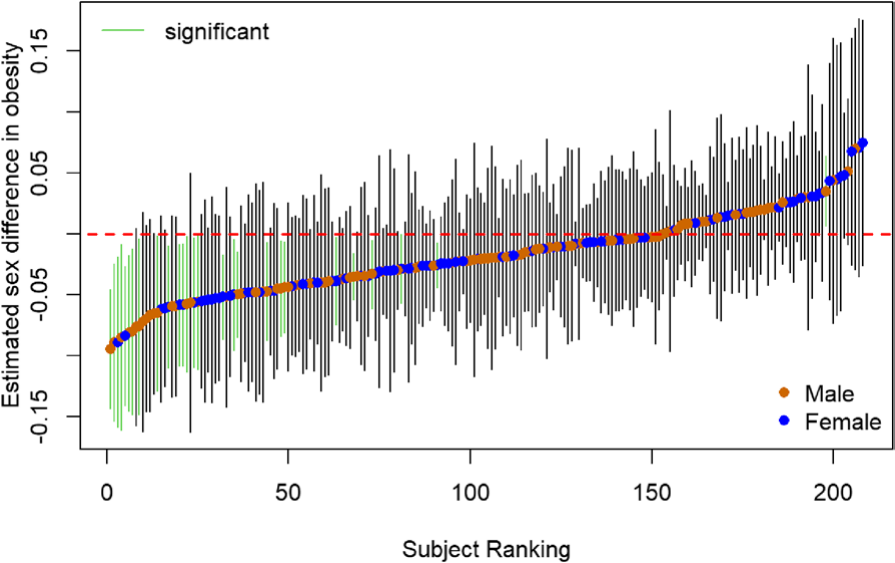
The estimated sex difference on obesity risk in personal prenatal environments. The personal prenatal exposure environments were defined by the mother’s dairy intake, cotinine levels, living richness facilities, and green spaces. The sex of the individual living in a particular prenatal environment is shown in blue (male) and orange (female). The bars show the 95%CI for the effect of a personal prenatal environment on females in relation to males. The intervals were estimated using causal modeling implemented in *teff*. Green lines are significant sex differences in obesity risk given by the prenatal environments

We asked whether a combination of the four significant exposures and their levels could define specific environments where one sex is likely more obese than the other one. The exposure residuals, adjusted by common covariates, were used in causal inference modeling, with the aim to classify individuals into environments of high sexual dimorphism in obesity. We considered the multiexposure profile defined by the mother’s dairy intake, cotinine levels, living richness facilities, and green spaces during pregnancy. We randomly selected a set of 208 individuals from the HELIX cohort to infer their expected sex difference in obesity risk given their personal multiexposure profiles. We thus applied the causal modeling algorithm *teff*, taking sex as the treatment variable, and observed 27 children (13 females, 14 males) living in personal environments where girls are less likely obese than boys. By contrast, we found only one boy living in a personal environment where girls are more likely obese than boys (Fig. 5).

**Fig. 6:**
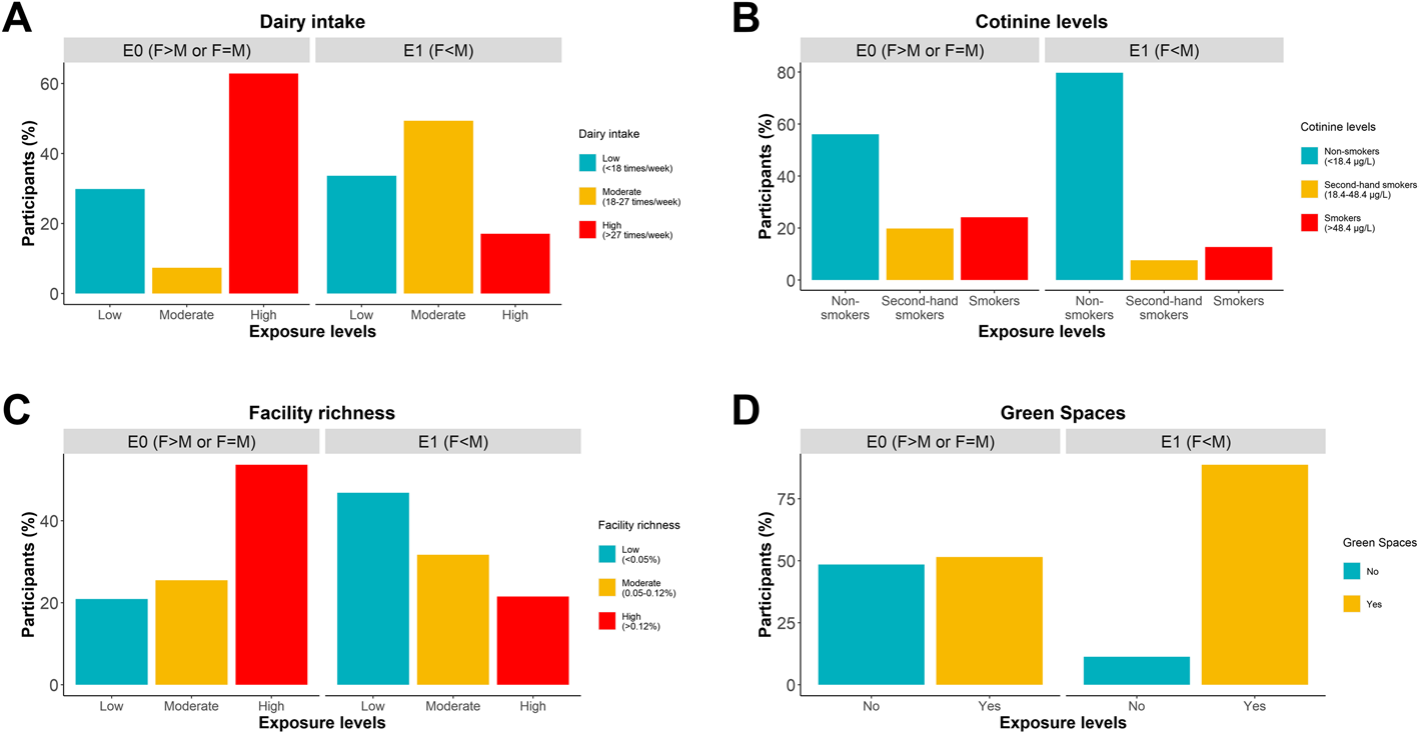
Characterization of the common prenatal environment where girls are more protected than boys against obesity (E1) against the reference environment (E0). Environment E1 is the common environment of individuals with personal environments where girls are significantly less obese than boys (female < male), these are the individuals with green confidence intervals in Fig. 5. E1 is defined by low mother dairy intake, non-smokers’ cotinine levels, low richness facilities, and the presence of green spaces. An individual belongs to E0 if he/she does not belong to E1 (female > male or female = male)

We aimed to classify all individuals into two environments: E1 and E0. The first one (E1) consisted of a combination of exposure levels that protects girls against obesity (F < M). The second one (E0) consists of the remaining combinations of exposure levels (F > M and F = M). E1 was obtained using the personal environments of the 27 children where girls are expected to be less obese than boys. E1 was defined as a binary vector, with one entry for each level of the four exposures, indicating whether a given exposure averaged across 27 individuals was higher or lower than the average across the entire training set of 208 children. We used the multiexposure profile to classify all the individuals in HELIX and observed a total of 675 (64%) individuals classified into E1. We found that E1 was characterized by moderate dairy consumption, non-smokers’ cotinine levels, low abundance of facility richness, and the presence of green spaces (Fig. 6A–D). Therefore, the environment captured both obesity protection for girls and obesity risk for boys, as expected from the individual exposures.

**Fig. 7:**
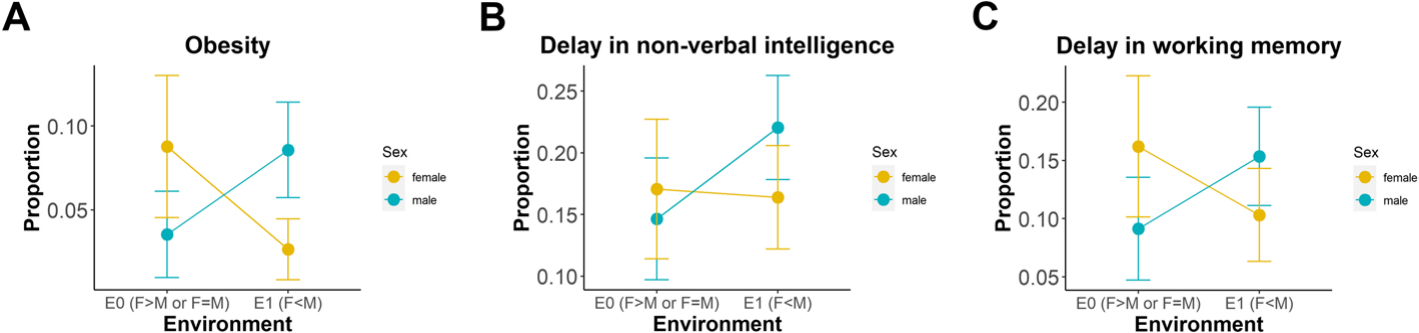
**A** Sex-environment interaction plot on obesity risk. The figure shows that E1 defines a prenatal environment across HELIX of strong female protection against childhood obesity (female < male), while E0 defines a prenatal environment without sexual dimorphism in obesity (F = M) or with male protection (F > M). **B** Sex-environment interaction plot on raven’s matrices underperformance. Affected individuals are those with outcomes below the first quintiles. **C** Sex-environment interaction plot on N-back underperformance. Affected individuals are those with outcomes below the first quintiles

We then observed a strong association of the sex-environment interaction on child obesity, adjusting by covariates (OR_interaction_ = 0.070, *P* = 2.59 × 10^−5^). Stratified associations by sex between the environment and obesity risk were also significant (girls: OR = 0.18, *P* = 4.73 × 10^−4^; boys: OR = 3.14, *P* = 0.012), suggesting stronger environment gains in the protection for girls than in the risk for boys (Fig. 7A). These results show that E1 can be regarded as a prenatal environment of female protection against childhood obesity, with much stronger protection than those given its individual exposure components.

### Sexual dimorphism in neurodevelopment

We asked whether the environment of high differences in obesity between sexes was also an environment of high differences in neurodevelopment. First, we assessed the association between obesity and four neuropsychological outcomes, fitting logistic regression models on obesity and adjusting by common covariates, sex, and the environment (E1/E0). We observed that low values of Raven’s matrices and N-back test tests were significant risk factors for obesity (OR = 2.42, *P* = 0.01; OR = 2.65, *P* = 0.02, see Fig. 7B), as ADHD diagnosis increased the risk (OR = 2.15, *P* = 0.03, see Fig. 7C). However, we did not find significant associations between obesity and attention outcome.

We tested whether the subject classification into the environments E1 and E0 significantly interacted with sex on each of the neuropsychological outcomes, as it did with obesity. We found that the sex-environment interaction was associated with higher outcomes of both Raven’s matrices (OR_interaction_ = 0.42, *P* = 0.047) and N-back test (OR_interaction_ = 0.31, *P* = 0.02), suggesting a higher performance of girls with respect to boys in these two tests, within E1. Associations were fully adjusted by covariates.

### Methylation profile associated with the prenatal environment of high sex differences in obesity

**Fig. 8:**
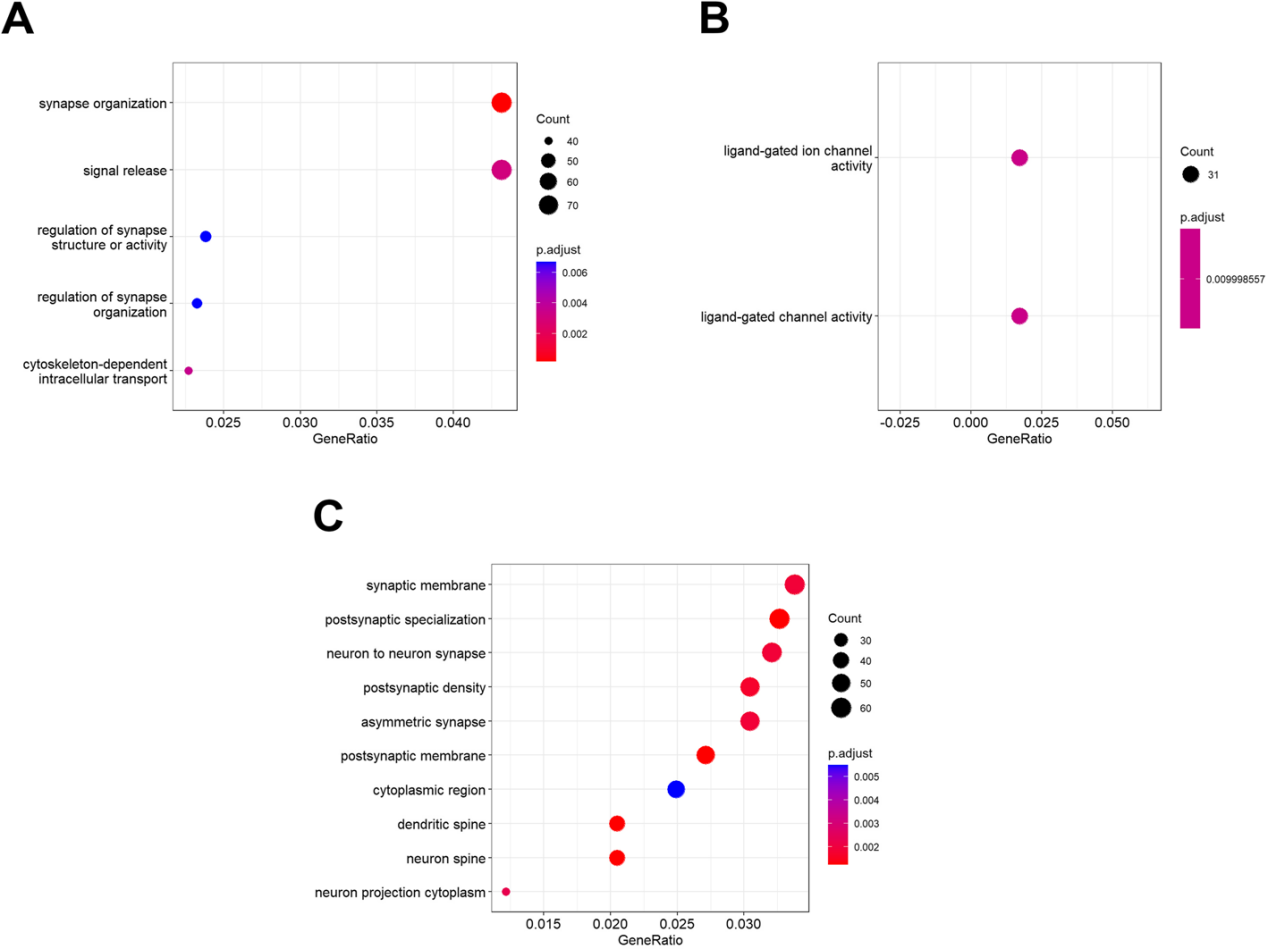
Enrichment analysis for the differentially methylated sites associated with E1. **A** Gene Ontology. **B** Cellular components. **C** Biological processes. Epigenome-wide analysis for the prenatal environment E1 was performed and methylation probes with associations at *P* < 0.001 were selected. Probes were mapped to genes that were used in enrichment analyses. The analyses mainly show pathways related to neuronal function

We aimed to investigate whether the methylome captured the differences between individuals belonging to E1 or E0. We performed an EWAS of the classification of children in the prenatal environment, adjusting by common covariates and immune cell counts. Methylation data was extracted from blood samples and were previously normalized and corrected for surrogate variation. We did not observe any significant association at a genome-wide level after correcting for multiple comparisons, see top associations in Additional file 2: Table S2. We also performed an enrichment analysis for the top associations (nominal *P* < 0.01). We tested different GO terms from molecular function, cellular components, and biological processes (Fig. 8), and observed several pathways related to neuronal processes. Most remarkably, *synapse organization* (*P*-adjusted = 0.0001) and *regulation of synapse structure or activity* (*P*-adjusted = 0.006) are two biological processes directly related to neurodevelopment.

## Discussion

We have shown in the HELIX cohort that environments defined by a multiexposure profile with different effects on obesity for each sex can be identified with the novel use of causal inference [10]. In a previous study on the same cohort, no significant associations were observed for individual prenatal exposures with overweight and obesity status, while cotinine levels were associated with BMI only at nominal significance [37]. Although we observed only four nominally significant interactions between prenatal exposures and sex on obesity, we revealed a prenatal environment defined by specific levels of these exposures whose effect on obesity strongly changed between sexes, with a 93% reduction in obesity risk for girls in relation to boys (OR_interaction_ = 0.070, *P* = 2.59 × 10^−5^). In the environment defined by moderate dairy consumption, non-smokers’ cotinine levels, low facility richness, and the presence of green spaces, girls are more protected than boys against obesity.

Previous studies have shown conflictive findings on dairy intake during pregnancy and its relation to long-term body composition of children. Voerman et al. reported significant associations with abdominal fat in children and strong interaction with sex on the pericardial fat mass index, with a higher risk for girls [38]. However, other studies have reported no significant associations [39, 40]. Our findings suggest that part of the discrepancy could be due to the interaction with sex.

Concerning obesity and cotinine levels in the blood of pregnant mothers, previous studies have shown a 50% increase in childhood overweight for smoking during pregnancy [41], with a dose–response relationship [42]. Cotinine levels have also been associated with low birth weight but rapid gains in BMI after delivery [43]. In a Japanese population, Susuki et al. observed that boys of mothers who smoked during pregnancy had higher gains in BMI trajectories compared with girls [44]. We found, however, higher obesity frequency for girls of mothers with smoker’s cotinine levels. In a large study of ~ 90,000 mother-children pairs, also in Japan [43], they observed that rapid gains in BMI of children were associated with urinary cotinine concentration of mothers but not with self-reported smoking status. While their results were not stratified by sex, it shows that cotinine is a more accurate assessment of pregnancy smoking.

In relation to green spaces, systematic reviews have shown weak evidence for its relationship with children’s obesity [45, 46]. Associations of green spaces during pregnancy and their differential effect on sex have not been previously assessed. We found that prenatal green space is a risk factor for boys’ obesity only. A recent study of the HELIX cohort showed significant associations between children’s overweight and obese status with the built environment (land use mix) [47]. Children living in built environments in absence of green spaces could be at higher risk of obesity (likely due to its relationship with physical activity). However, we observed that a low abundance of facility richness and the presence of green spaces during pregnancy are risk factors for obesity in boys. Both environmental conditions of the pregnant mother are consistent with less urbanized environments where adult obesity may be more frequent [48].

In this study, we observed that the combination of the specific levels for the four exposures maximizes the differences in obesity risk between girls and boys. Previous studies have already suggested that better prediction of an outcome can be obtained from the aggregation of multiple environmental factors into risk scores [49, 50] or the use of mixture models [51]. In line with this, we used causal inference for classifying the individuals in two environments (E0 and E1) based on the combination of the four exposures.

After classifying individuals in the two environments, we further investigated whether the individuals belonging to the environment with higher sexual dimorphism in obesity presented also sexual dimorphism in neurodevelopmental delay. Based on previous studies, prenatal factors, such as maternal obesity, have been seen associated with both obesity in children and lower cognitive abilities and ADHD [52, 53]. Animal studies have shown that mice whose mothers were on high-fat diets during pregnancy have alterations in brain methylation of dopaminergic and opioid genes [54, 55]. In addition, the neurodevelopmental delay appears to be more frequent in obese boys [12]. A longitudinal prospective study has shown that working memory and attention performance are reduced by increasing BMI in children [56]. Our study offers additional evidence of this relationship, since the environmental changes that modulate the association between sex and obesity also modulate the association between sex and neurodevelopmental delay. Furthermore, the environment is associated with methylation probes that are enriched in neurodevelopmental pathways, providing more evidence for this hypothesis.

The generalizability of these results is subject to certain limitations. First, we did not observe a significant sexual dimorphism in obesity as expected. In the HELIX population, 4.9% of girls were obese contrasting with 6.8% of boys. Since we found that there is a clear difference, we suspect that it was not significant due to the low number of children with obesity (39 boys and 23 girls). By contrast, we found significant differences between the sexes for the ADHD diagnosis and the ANT distribution. In this case, the number of children affected was higher than in obesity (77 boys and 27 girls for ADHD and 134 boys and 72 girls for ANT). Second, the interactions between prenatal exposures and sex in obesity were significant at a nominal level but not after correcting by multiple comparisons. Again, this could be because of the small sample size and the low statistical power, which is especially important when evaluating interactions.

Our study also had notable strengths. We confirmed that the combination of exposures greatly increased the significance of interactions between prenatal exposures and sex. We also confirmed the importance of the relationship between the obesogenic prenatal environment and neurodevelopment. We not only found a significant sexual dimorphism in neurodevelopment delay when comparing E1 and E0, but we also found enrichment in neurodevelopmental pathways in the methylation probes associated with the environment. This provides a possible molecular mechanism that could explain the association between the obesogenic environment and sexual dimorphism in neurodevelopment. Moreover, this study evaluates the different effects of a prenatal environment in girls and boys, which is very innovative and important to consider sex differences in the prenatal exposure guidelines.

## Conclusions

We aimed to advance a novel approach to the study of sexual dimorphism, based on high dimensional exposure data and recent methods of causal inference. The methodological approach can also be used to determine the environmental landscape that promotes sexual dimorphisms in studies with high dimensional exposure data.

In summary, girls in childhood may be protected against obesity if their pregnant mothers had moderate dairy consumption, non-smokers cotinine levels, and lived in environments with a low abundance of rich facilities and the presence of green spaces. The environment is also protective against the neurodevelopmental delay of non-verbal intelligence and working memory. While female protection is measured against male risk, female protection outweighs the risk of obesity in boys. Our study motivates further public health efforts to raise public awareness of moderating a high-fat diet and avoiding smoking and second-hand smoking during pregnancy to protect children against obesity and neurodevelopmental delay.

## Supplementary Information

**Additional file 1. **Supplementary Methods.

**Additional file 2: Table S1.** Exposure variables in the HELIX cohort. **Table S2.** CpG sites with a nominal *P*-value lower than 0.001 in the epigenome-wide association study for the classification of children in the prenatal environment (E0 and E1).

**Additional file 3.** Supplementary Code.

## Data Availability

Any custom code or software used in our analysis is available at Additional file 3: Supplementary Code. The HELIX data warehouse has been established as an accessible resource for collaborative research involving researchers external to the project. Access to HELIX data is based on approval by the HELIX Project Executive Committee and by the individual cohorts. Further details on the content of the data warehouse (data catalog) and procedures for external access are described on the project website (http://www.projecthelix.eu/index.php/es/data-inventory). The data used in this analysis are not available for replication because specific approvals from the HELIX Project Executive Committee and the University of Southern California Institutional Review Board must be obtained to access them.
