# Supplementary material for: Prenatal environmental exposures associated with sex differences in childhood obesity and neurodevelopment
Source: BMC Med. 2023 Apr 12;21:142. doi: 10.1186/s12916-023-02815-9 (PMC10099694; doi:10.1186/s12916-023-02815-9)
Supplement: Supplementary file 1 — Additional file 1. Supplementary Methods. [file 12916_2023_2815_MOESM1_ESM.docx]

**Prenatal environmental exposures associated with sex differences in childhood obesity and neurodevelopment**

**Additional File 1: Supplementary Methods**

## Exposome assessment in pregnancy

Two main windows of exposure were considered, one cross-sectional including the exposome data of children at the same time as of omics sampling (childhood), and a prenatal window including the pregnancy period or measures of long-term maternal exposures (e.g., persistent pollutants). A total of 91 prenatal and 116 childhood exposures were investigated in the study, including the outdoor exposome (air pollution, built environment, noise, green and blue space, and meteorological data), the individual exposome (cotinine, metals, POPs, PFAS, phthalates, phenols, and organophosphates) as well as lifestyle factors (exposure to tobacco smoking, diet and physical activity). They were measured in diverse ways as described below.

The outdoor exposome was assessed through GIS information and existing land use regression models adjusted for data from regulatory monitors and remote sensing data [20]. In this particular study, we analyzed whole pregnancy levels of air pollution at the home address (the year average before follow-up), while first pregnancy trimester levels of meteorological variables. Built environment was calculated in a 300 m buffer.

Biomarkers of chemical exposures were measured in samples from mothers during pregnancy. Biomarkers include: organochlorine compounds (OCs) and brominated compounds (PBDEs) in serum, perfluoroalkyl substances (PFASs) in plasma, metals and essential minerals in whole blood, and non-persistent chemicals (phthalates, phenols, organophosphate pesticides (OPs), and cotinine in urine samples. Further details can be found elsewhere [27].

Lifestyle factors were assessed through standardized questionnaires: including the KIDMED questionnaires to assess Mediterranean diet [28], socioeconomic status (family affluence scale [29], and subjective wealth), social capital of the family [30], exposure to environmental tobacco smoke, water consumption habits, cooking and heating methods at the home, cleaning products, noise perception, and use of green spaces. Concentrations of drinking water disinfection by products (DBPs) during pregnancy were estimated from water company concentration and habits obtained from questionnaire data. Indoor air concentrations of nitrogen dioxide (NO2), particulate matter <2.5μm (PM2.5), particulate matter absorbance (PMabs), benzene, and toluene, ethylbenzene, xylene (BTEX) were estimated through a prediction model that combined measurements in the homes of a subgroup of children with questionnaire data from the subcohort.

Exposures were either continuous variables or categorical variables with two or more levels. Continuous exposure variables were transformed to achieve linearity or categorized, when needed. Missing data were imputed using a chained equations method [31] implemented in the mice v3.4.0 R package [32]. Twenty imputed datasets were created, although we only used the first imputation in this study. The correlation among exposures, within each exposure window or overall has been described in detail elsewhere [33].
