# Supplementary material for: Prenatal environmental exposures associated with sex differences in childhood obesity and neurodevelopment
Source: BMC Med. 2023 Apr 12;21:142. doi: 10.1186/s12916-023-02815-9 (PMC10099694; doi:10.1186/s12916-023-02815-9)
Supplement: Supplementary file 3 — Additional file 3. Supplementary Code. [file 12916_2023_2815_MOESM3_ESM.pdf]

Prenatal environmental exposures associated with sex differences in childhood obesity and neurodevelopment

Alejandro Cáceres, Natàlia Carreras-Gallo, Sandra Andrusaityte, Mariona Bustamante, Ángel Carracedo, Leda Chatzi, Varun B. Dwaraka, Regina Grazuleviciene, Kristine Bjerve Gutzkow, Johanna Lepeule, Léa Maitre, Tavis L. Mendez, Mark Nieuwenhuijsen, Remy Slama, Ryan Smith, Nikos Stratakis, Cathrine Thomsen, José Urquiza, Hannah Went, John Wright, Tiffany Yang, Maribel Casas, Martine Vrijheid, Juan R. González

Instituto de Salud Global de Barcelona (ISGlobal), Barcelona, Spain

Supplementary code

Our analysis uses only R/Bioconductor packages and our implementation of the random causal forest package teff (https://github.com/teff-package/teff), also in R.

```
library(ggplot2)
library(ggthemes)
library(ggpubr)
library(arm)
library(limma)
library(clusterProfiler)
library(minfi)
library(teff)
library(rexposome)
library(caret)
library(gridExtra)
library(metafor)
```

Prenatal exposure data

Permission was granted for the use of prenatal exposome data from the HELIX project (https://www.projecthelix.eu/ (https://www.projecthelix.eu/)). Data were downloaded into the local directory `./data` , from which they were loaded into the R session. We first inspected the exposome data

```
load("./data/Imppreg_final.Rdata")

phenotype <- pData(imppreg_final)
exposome <- expos(imppreg_final)
code <- fData(imppreg_final)
```

Annotation data for exposures were retrieved and written in `TableS1.txt` . Here we show the first six rows of the table

```
pregtable <- code[names(exposome),c("Exposure", "Description", "Group", "Transformation", "Missing_Per")]

write.table(pregtable, file="TableS1.txt", sep="\t", row=TRUE, col=TRUE, quote=FALSE)

head(pregtable)
```

	**Exposure** <chr>
e3_alcpreg_yn_None	e3_alcpreg_yn_None
e3_asmokyn_p_None	e3_asmokyn_p_None
h_abs_ratio_preg_Log	h_abs_ratio_preg_Log
h_accesslines300_preg_dic0	h_accesslines300_preg_dic0
h_accesspoints300_preg_Log	h_accesspoints300_preg_Log
h_blueyn300_preg_None	h_blueyn300_preg_None

6 rows | 1-2 of 6 columns

We show the bar plot of the exposures by their exposure families.

```
tb1 <- table(pregtable$Group)



cols <- names(tb1)
o <- order(cols)
cols <- cols[o]

b <- barplot(tb1, horiz = FALSE,las=2, ylab="Number of exposures", cex.names=0.3, col=rainbow(17)[as.numeric(factor(cols))], xaxt = "n", main="Pregnancy exposome (HELIX)")



text(x = as.vector(b),  y =par("usr")[3]-0.01 ,
     ## Use names from the data list.
     labels = names(tb1),
      xpd = NA,
      srt = 35,
      cex=0.5,
      adj=0.965
)
```

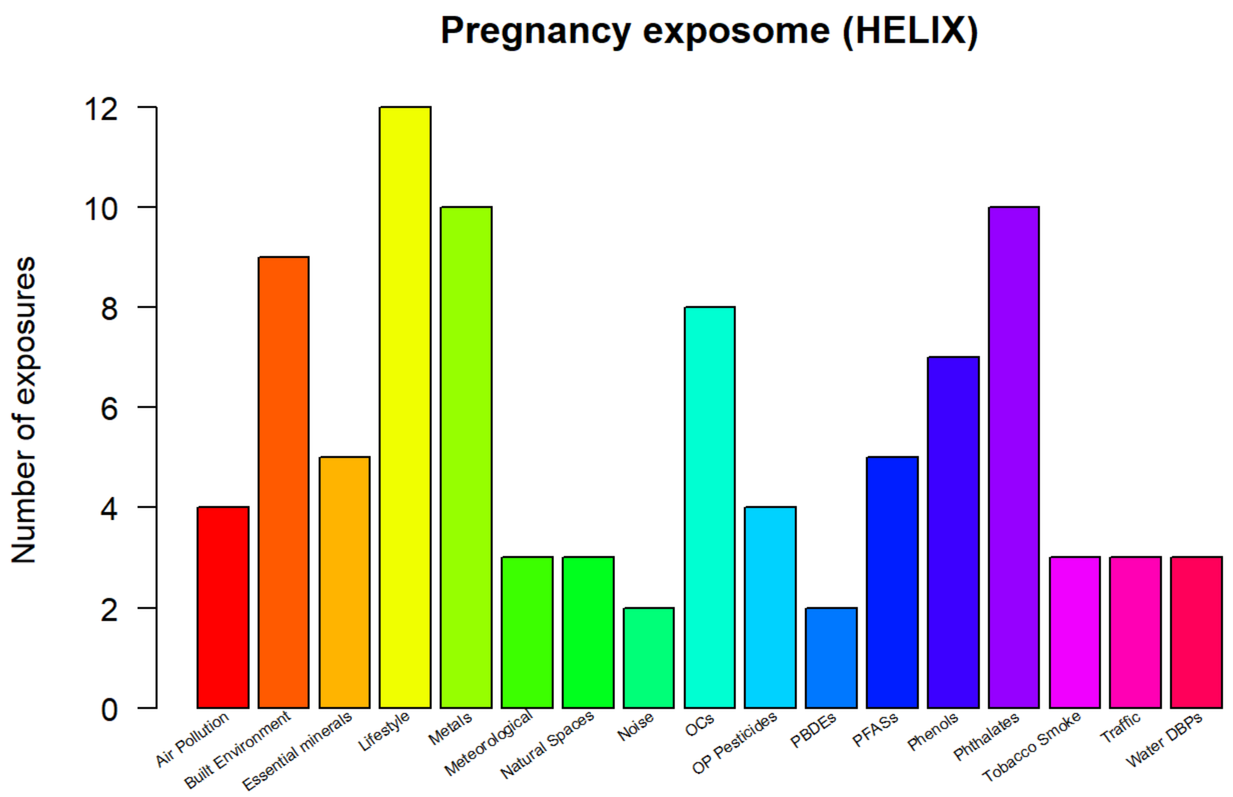

Phenotype data

We considered BMI and its derived categories, adjusted by age and sex.

```
par(mfrow=c(1,2))
hist(phenotype$hs_c_bmi_None, main="BMI", xlab="")

b <- barplot(table(phenotype$hs_bmicat_None), main ="BMI categories", xlab="", xaxt = "n")

text(x = as.vector(b),  y =par("usr")[3]-0.01 ,
     labels = names(table(phenotype$hs_bmicat_None)),
      xpd = NA,
      srt = 35,
      cex=0.5,
      adj=0.97
)
```

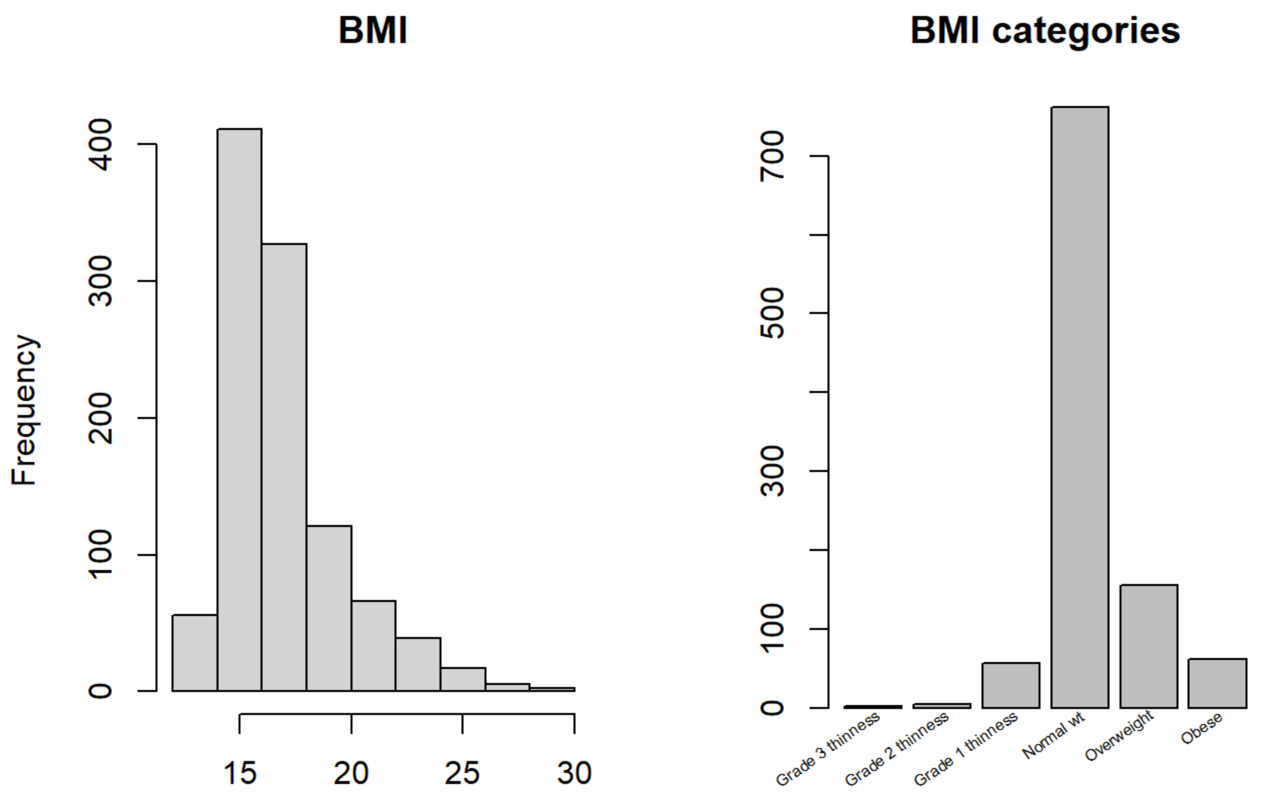

We considered obesity as the main outcome derived from the BMI categories.

```
table(phenotype$hs_bmicat_None)
```

```
##
## Grade 3 thinness Grade 2 thinness Grade 1 thinness        Normal wt
##                2                5               57              762
##       Overweight            Obese
##              156               62
```

```
phenotype$hs_bmicat_None <- as.numeric(phenotype$hs_bmicat_None%in% "Obese")
```

Main neurodevelopment outcomes were obtained from neuropsychological test batteries of working memory (N-back), attention (ANT) and non-verbal intelligence (Raven’s colored progressive matrices). We also considered ADHD status.

```
neuro <- read.delim("./data/neuro_helix_jr.csv", sep=",")
rownames(neuro) <- neuro$HelixID
neuro <- neuro[rownames(phenotype),]

phenotype$hs_correct_raven <- neuro$hs_correct_raven
phenotype$hs_accuracy_numeros2 <- neuro$hs_accuracy_numeros2
phenotype$hs_ADHD_raw_Cat <- neuro$hs_ADHD_raw_Cat
phenotype$hs_accuracy_ANT <- neuro$hs_accuracy_ANT

par(mfrow=c(2, 2))
hist(phenotype$hs_correct_raven, main="Raven's matrices", xlab="")
barplot(table(phenotype$hs_ADHD_raw_Cat), main= "ADHD")
hist(phenotype$hs_accuracy_numeros2, main="N-back (2-back accuracy)", xlab="")
hist(phenotype$hs_accuracy_ANT, main="ANT (accuracy)", xlab="")
```

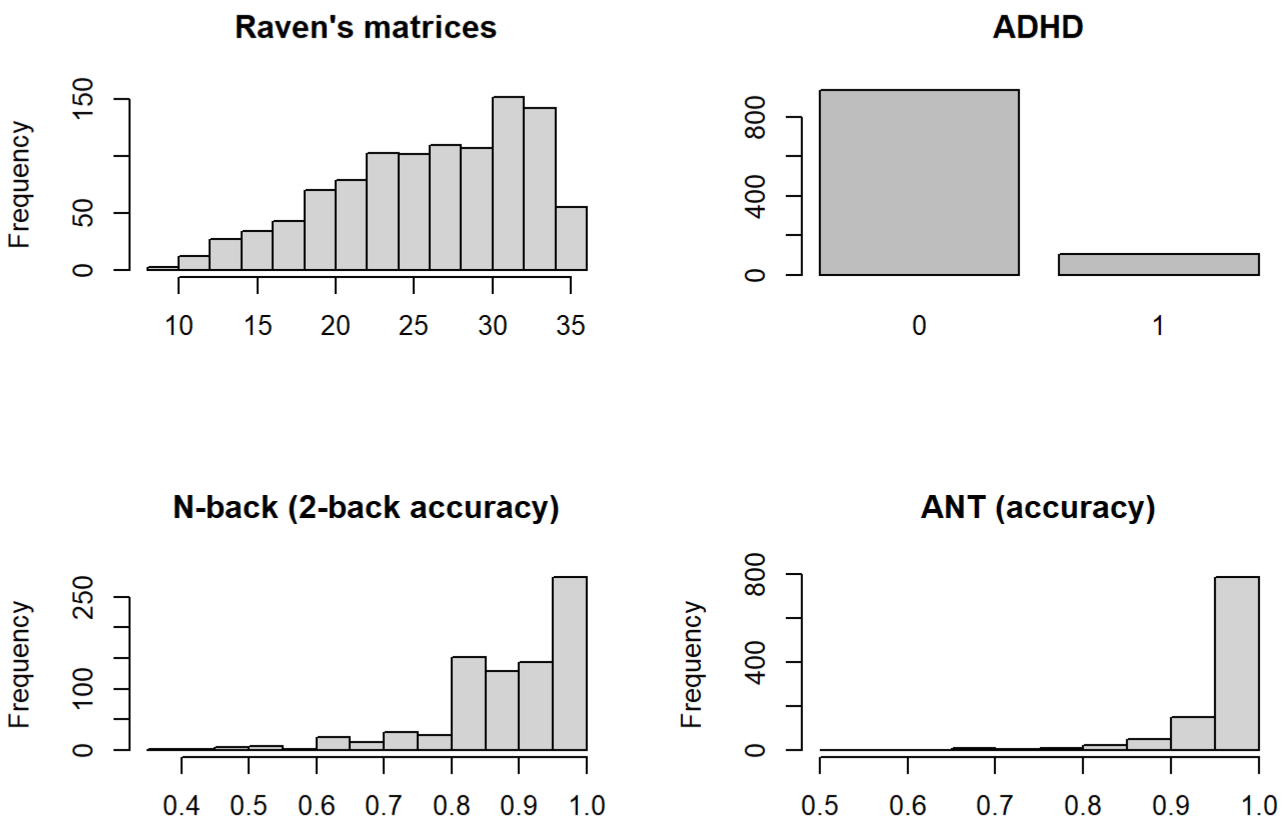

We dichotomized the neuropsychological outcomes, taking as cases individuals with outcomes below the first quintiles (20%). We thus studied the clinical events of dropping below the first quintile for different neurodevelopment features.

```
#raven
phenotype$hs_correct_raven <- as.numeric(phenotype$hs_correct_raven < quantile(phenotype$hs_c
orrect_raven, 0.20, na.rm=TRUE))

table(phenotype$hs_correct_raven)
```

```
##
##   0   1
## 850 189
```

```
#n-back
phenotype$hs_accuracy_numeros2 <- as.numeric(phenotype$hs_accuracy_numeros2 < quantile(phenotype$hs_accuracy_numeros2, 0.20, na.rm=TRUE))


table(phenotype$hs_accuracy_numeros2)
```

```
##
##   0   1
## 705 104
```

```
#ant
phenotype$hs_accuracy_ANT <- as.numeric(phenotype$hs_accuracy_ANT < quantile(phenotype$hs_accuracy_ANT, 0.20, na.rm=TRUE))

table(phenotype$hs_accuracy_ANT)
```

```
##
##   0   1
## 823 206
```

```
#adhd
table(phenotype$hs_ADHD_raw_Cat)
```

```
##
##   0   1
## 933 104
```

We explored the extent to which the clinical outcomes were sexually dimorphic. We assessed their association with sex by a linear model for BMI and logistic linear regression models for dichotomic outcomes. We took males as the reference category; and adjusted by cohort, year of birth, mother’s BMI, mother’s weight gain during pregnancy, gestational age, mother’s age at pregnancy, mother’s education, whether parents were native from the country cohort, parity and children age at clinical assessment.

```
covs <- c("cohort", "e3_yearbir_None",   "h_mbmi_None", "hs_wgtgain_None", "e3_gac_None",
"h_age_None", "h_edumc_None","h_native_None", "h_parity_None", "hs_child_age_days_None")



nms <- c("hs_bmicat_None", "hs_correct_raven", "hs_accuracy_numeros2", "hs_accuracy_ANT", "hs
_ADHD_raw_Cat")

phenotype$sex <- relevel(phenotype$sex,"male")

#dimorphism adjusted

dims <- lapply(nms, function(ph)
{
  mod <- "gaussian"

  y <- phenotype[[ph]]
  sex <- phenotype$sex
  cohort <- phenotype$cohort
  covmat <-   phenotype[,covs]


   phenored <- data.frame(y=y, sex=sex, covmat)


  fa <- formula(paste0("y~", paste0(names(phenored)[-1],
                                  collapse="+")))




  if(length(table(y))==2){
     mod <- "binomial"
  }

  summary(glm(fa, family = mod, data = phenored))
})

sexassoc <- t(sapply(dims, function(x) x$coefficients["sexfemale", c(1,2,4)]))
rownames(sexassoc) <- nms

sexassoc[,c(1,2)] <- exp(sexassoc[,c(1,2)])

sexassoc
```

```
##                       Estimate Std. Error     Pr(>|z|)
## hs_bmicat_None       0.6410240   1.341731 1.303433e-01
## hs_correct_raven     0.7216166   1.220969 1.022156e-01
## hs_accuracy_numeros2 0.9381181   1.262961 7.843749e-01
## hs_accuracy_ANT      0.5371198   1.193150 4.323602e-04
## hs_ADHD_raw_Cat      0.3664449   1.271248 2.877319e-05
```

Exposure environment of high obesity differences

between sexes

We first assessed the effect of all the interactions between sex and prenatal exposures on obesity, using logistic regressions adjusted by covariates. We selected the exposures that had significant interactions with sex, at a nominal level. We found four exposures, namely:

- Dairy consumption during pregnancy (times/week): Categories: < 17.1, $17.1 - 27.1$ and > 27.1
- Number of different facility types present divided by the maximum potential number of facility types (at a 300m buffer)
- Green spaces (Is there a greenspace whithin a distance of 300m?)
- Categorical variable of cotinine in mother. Categories: Non-smokers (< 18.32), SHS smokers ($18.4 - 48.4$) and Smokers (> 50).

```
ph <- "hs_bmicat_None"

texp <- lapply(names(exposome), function(xx){
  dat4int <- data.frame(t = as.numeric(phenotype$sex )-1,
                        eff = phenotype[,ph],
                        exp = as.numeric(exposome[,xx]),
                        phenotype[covs])


  fa <- formula(paste0("eff ~ t:exp +",
                       paste0(names(dat4int)[-2], collapse="+")))

  modint <- summary(bayesglm (fa, data=dat4int, family="binomial"))$coeff["t:exp", c(1,4)]


  fa <- formula(paste0("eff ~ ",
                       paste0(names(dat4int)[-c(1,2)], collapse="+")))


  modmale <- summary(bayesglm (fa, data=dat4int[dat4int$t==0,],  family="binomial"))$coeff["e
xp", c(1,4)]

  modfemale <- summary(bayesglm (fa, data=dat4int[dat4int$t==1,],  family="binomial"))$coeff
["exp", c(1,4)]

  fa <- formula(paste0("eff ~ ",
                       paste0(names(dat4int)[-2], collapse="+")))

  mod <- summary(bayesglm (fa, data=dat4int, family="binomial"))$coeff["exp", c(1,4)]

  data.frame("sex-exp"=modint[1],"P"=modint[2],
             "exp|male"=modmale[1], "P|male"=modmale[2],
             "exp|female"=modfemale[1],"P|female"=modfemale[2],
             check.names = FALSE, "exp"=mod[1],"P"=mod[2])

})

texp <- do.call(rbind, texp)
rownames(texp) <- names(exposome)

nmssig <- names(exposome)[texp[,2]<0.05]

texpsig <- texp[texp[,2]<0.05,]

dd <- data.frame(labels=code[nmssig,"LabelTable"], texpsig, check.names = FALSE)

dd <- dd[order(dd[,3]),]

rownames(dd)<- NULL

dd[,c(2,4,6,8)] <- exp(dd[,c(2,4,6,8)])
dd
```

labels <chr>	sex-exp <dbl>	P <dbl>	exp|male <dbl>	P|male <dbl>	exp|female <dbl>
Dairy intake	2.4465716	0.008144587	1.0418203	0.860912277	2.8820535
Facility richness (300m)	1.1059144	0.013391939	0.9182055	0.005171934	1.0347706
Green spaces (300 m)	0.2797034	0.029674630	5.0655255	0.007279687	0.6406668
Cotinine	1.9234787	0.034222247	0.8290350	0.424215847	1.9139793

4 rows | 1-7 of 9 columns

```
cols <- code[rownames(texp),"Group"]
o <- order(cols)
texp <- texp[o,]
cols <- cols[o]

tb <- table(cols)

pchs <- unlist(lapply(1:17, function(x) if(x %in% seq(1,17,2)) rep(16, tb[x]) else rep(18, tb
[x])))

plot(-log10(texp[,2]), pch=pchs, ylab="-log P", xlab="Exposures", col=rainbow(17)[as.numeric
(factor(cols))], main="Asocciation of obesity in childhood with \n the interactions of Sex an
d Prenatal Exposures", ylim=c(0,3))

abline(h=-log10(0.05), lty=2)

legend("topright", legend=names(tb), col=rainbow(17), pch=rep(c(16,18),11)[-18], horiz = FALS
E, cex=0.36, bty = "n")

for(i in 1:nrow(texpsig))
text(which(rownames(texp)==rownames(texpsig)[i]), -log10(texpsig[i,2]), dd[i, "labels"] , cex
=0.7, pos=1)
```

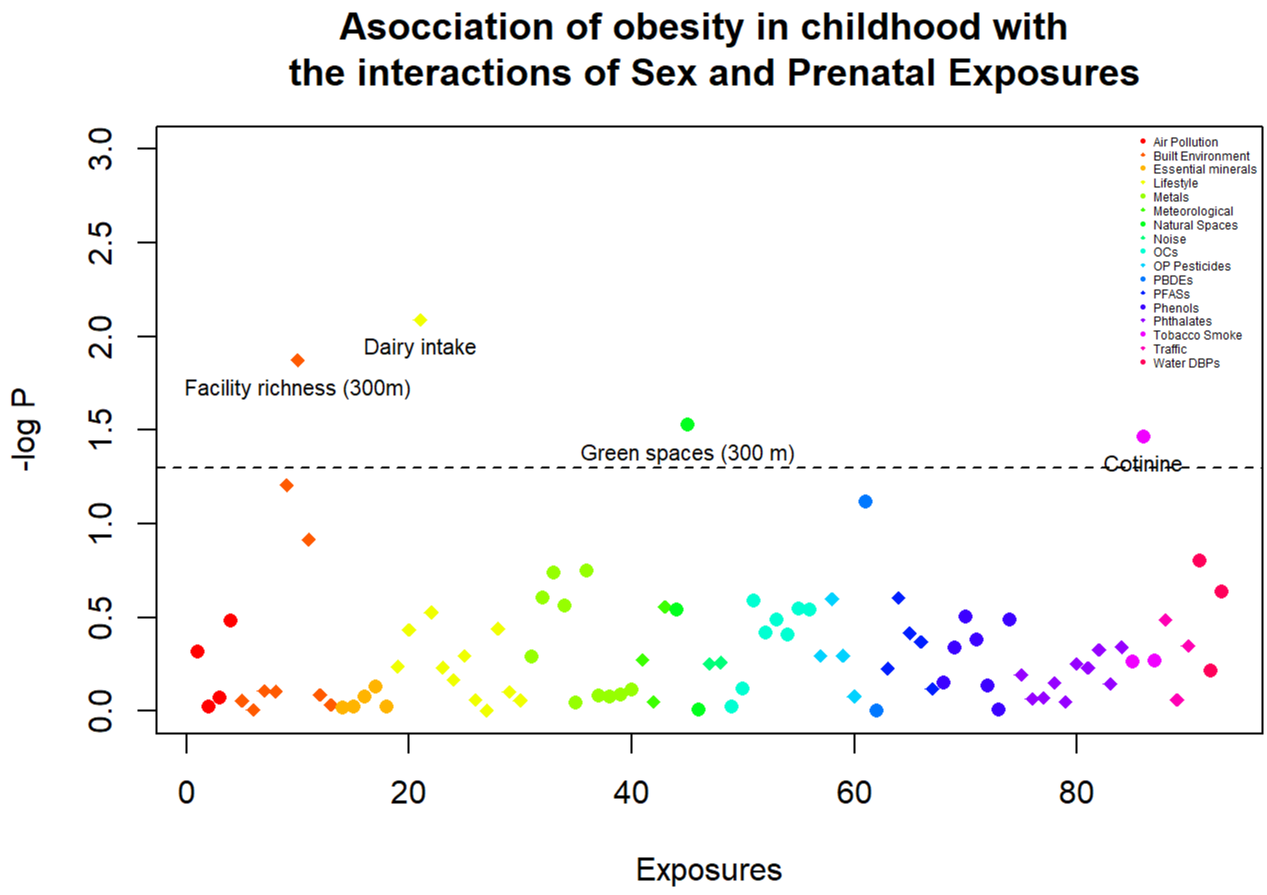


```
## png
##   2
```

We asked whether a combination of these exposures could define personal environments where females are expected to be more obese than males. The exposure residuals, adjusted by covariates, were used in causal inference modeling, with the aim to classify individuals into environments of high sexual dimorphism in obesity. We therefore applied the algorithm `teff` taking sex as the treatment variable (https://teff-package.github.io/ (https://teff-package.github.io/)).

We observed several individuals in personal environments with likely high sexual dimorphism in obesity. However, we only found personal environments with significant protection for female obesity, and not for the risk of female obesity.

```
phenored <- data.frame(eff = phenotype[,ph],
                       t = as.numeric(phenotype$sex)-1,
                       phenotype[covs])

names(phenored)[3:ncol(phenored)] <- paste0("cov", 3:ncol(phenored))


fa <- formula(paste0("~", paste0(names(phenored),
                                 collapse="+")))
mod <- model.matrix(fa, data = phenored)[,-1]

fa <- formula(paste0("~", paste0(names(exposome),
                                 collapse="+")))
modexp <- model.matrix(fa, data = exposome)[,-1]


data4teffob <- list(features=data.frame(modexp), teffdata = data.frame(mod))

finf <- unlist(sapply(nmssig, function(x) grep(x, colnames(data4teffob$features))))

finf <- colnames(data4teffob$features)[finf]


predob <- predicteff(data4teffob, featuresinf = finf)

ctrl <- list(lb=c("Male", "Female"), wht="bottomright", whs = "topleft")
plotPredict(predob, lb="Estimated sex difference in obesity", ctrl.plot=ctrl)
```

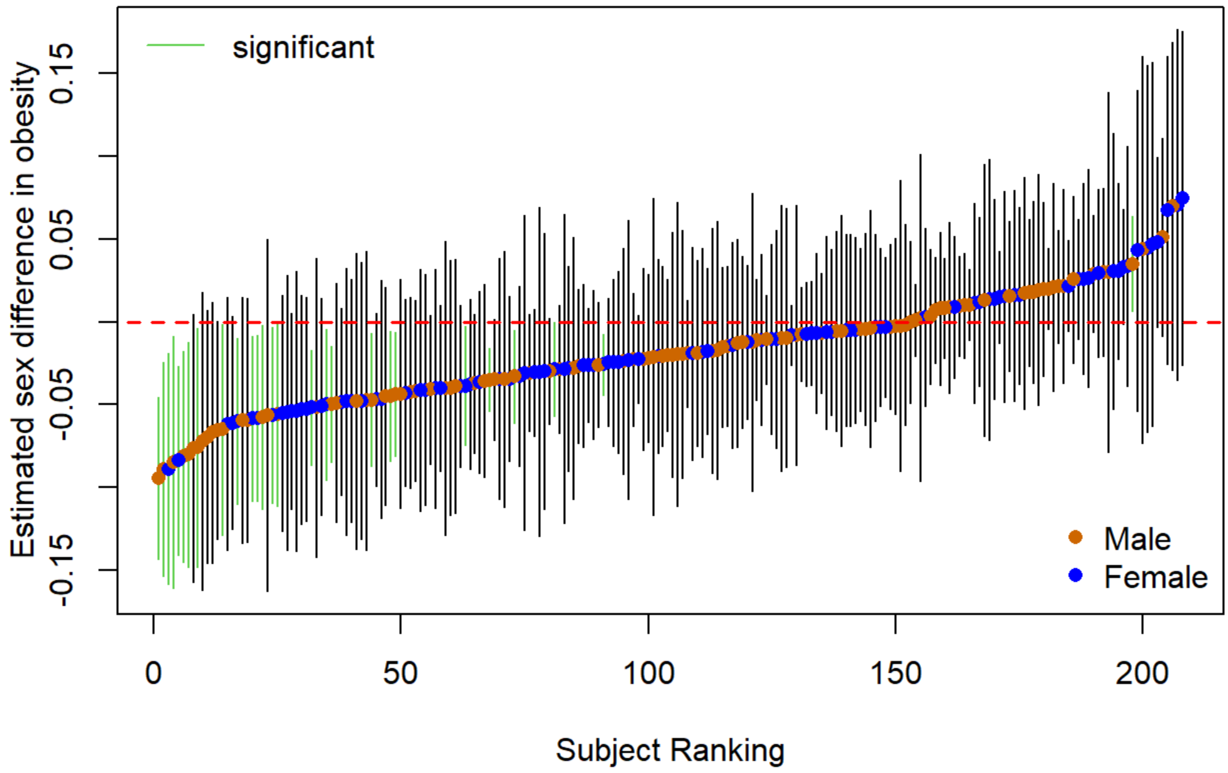

We estimated the common environment of personal environments girls are likely less obese than boys. We thus set the parameter profile to TRUE in the `predicteff` function. We reclassified all the subjects in the study according to their match in the common environment using the function `target`.

We confirmed in the whole dataset that in the inferred common environment girls are significantly more protected against obesity, while boys are at an increased risk (E1; F <M). We tested the associations of the environment with obesity, first stratified and then modulated (interaction) by sex.

```
predob <- predicteff(data4teffob, featuresinf = finf, profile = TRUE)
tarob <- target(data4teffob, predob, effect = "negative",
              model = "binomial",  match=0.6,
              lb=c("dairy_Ter2", "dairy_Ter3", "richness", "greenspace", "cotinine_Ter2","cot
inine_Ter3"))
```

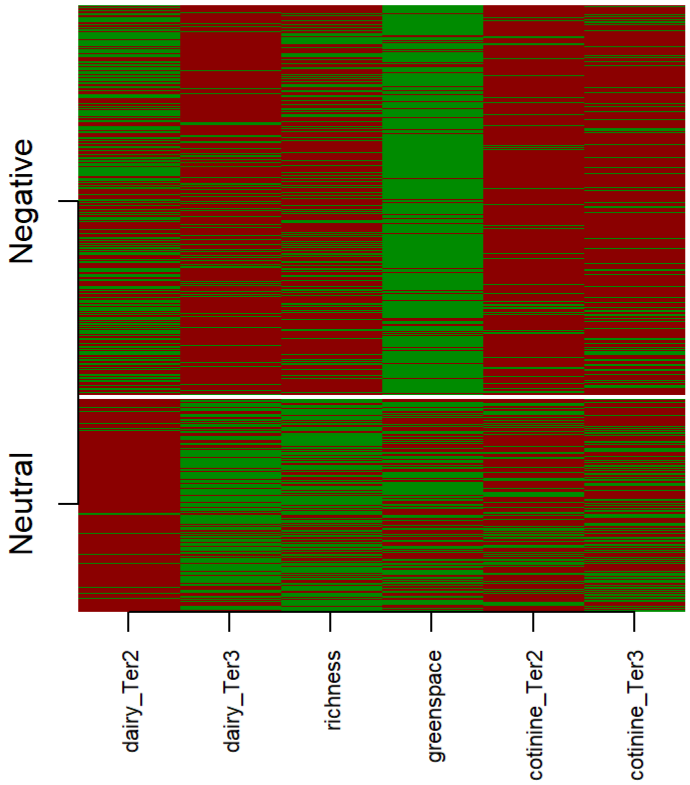


```
tarob
```

```
## object of class: tarteff
##
## classification into
##   negative treatment effect: 1
##   neutral: 0
##
##   0   1
## 369 675
##
## interaction fitted model: binomial
##      Estimate    Std. Error       z value      Pr(>|z|)
## -2.2025491239  0.6194770921 -3.5554972929  0.0003772648
```

```
y <- phenotype[[ph]]
expenv <- tarob$classification[rownames(phenotype)]
sex <- phenotype$sex
cohort <- phenotype$cohort
covmat <-   phenotype[,covs]


phenored <- data.frame(y=y, expenv=expenv, sex=sex, covmat)




#code["h_greenyn300_preg_None","Categories"]
datplot1 <- data.frame(ob = factor(phenotype[,ph],
                                    labels=c("Normal", "Obese")),
                      exposure = factor(tarob$classification[rownames(phenotype)],
                                      labels=c("E0 (F>M)", "E1 (F<M)")),
                      sex=phenotype$sex)


pls <- list()
ss <- 0
for(pf in c("E0 (F>M)", "E1 (F<M)")){
  dt <- datplot1[datplot1$exposure==pf,]
   pls[[ss+1]] <-ggplot(dt, aes(ob, fill = sex)) +
    geom_bar(position = "fill") +
    scale_y_continuous(labels = scales::percent)+
    labs(x = "", y="", fill = "Sex")+
    ggtitle(pf)

   ss <- ss+1
}

grid.arrange(pls[[1]], pls[[2]], nrow = 1)
```

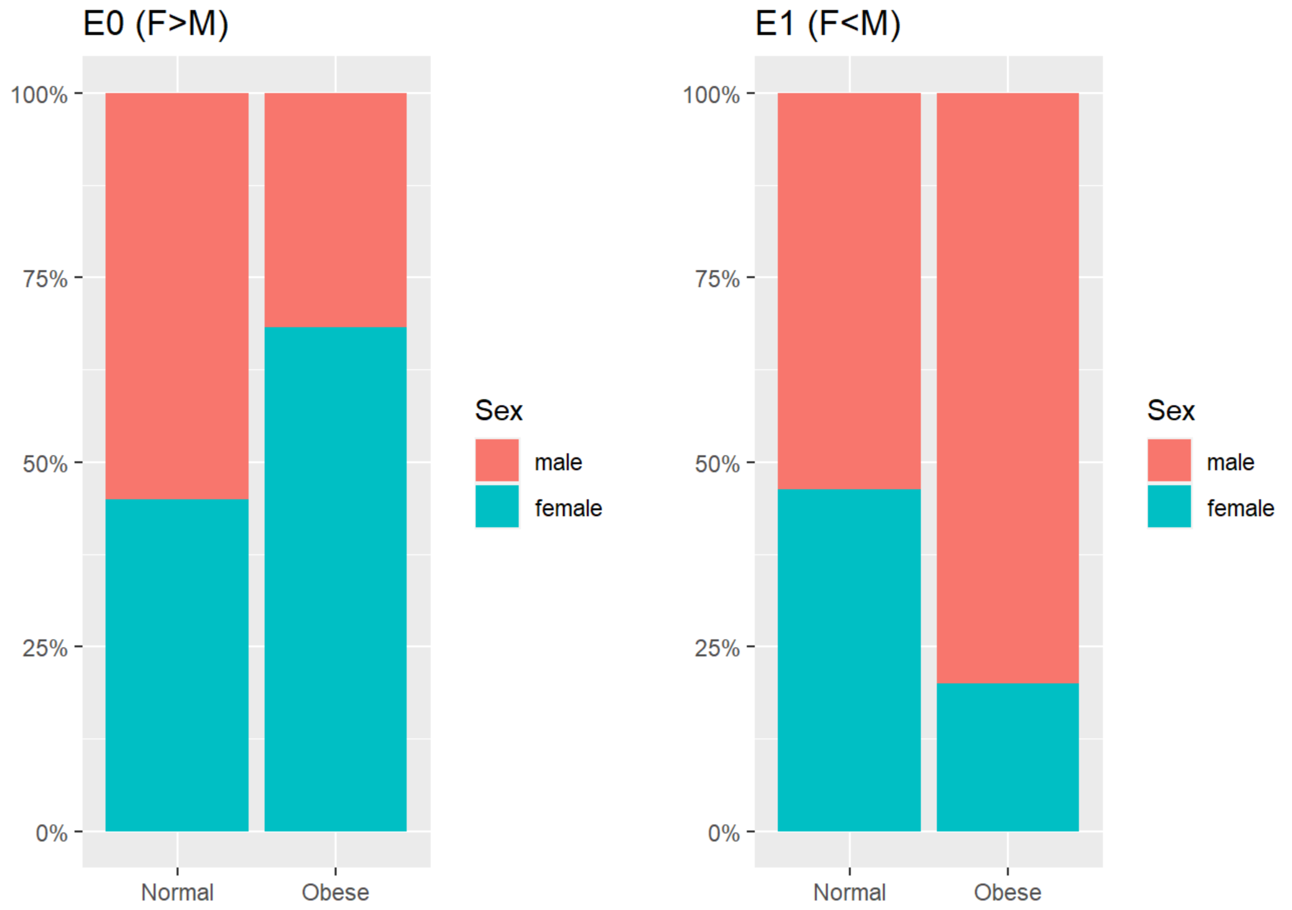


```
###
# interaction adjusted by covariates
phenored$sex <- as.numeric(phenored$sex)-1

fa <- formula(paste0("y~expenv:sex+", paste0(names(phenored)[-1],
                                  collapse="+")))

mod <- summary(bayesglm(fa, family = "binomial", data = phenored))$coefficients["expenv:sex",
c(1,4)]

mod[1] <- exp(mod[1])

mod
```

```
##     Estimate     Pr(>|z|)
## 7.081955e-02 2.591912e-05
```

We performed a meta-analysis of the sex-E1 interaction across cohorts, to confirm that the effect was consistent and homogeneous across studies.

```
###meta-analysis by cohort

modcohort <- lapply(levels(phenored$cohort), function(cc){
  fa <- formula(paste0("y~expenv:sex+",
                       paste0(names(phenored)[-c(1,4)],
                              collapse="+")))
  res <- summary(bayesglm(fa, family = "binomial",
                          data = phenored[phenored$cohort==cc,])
                 )$coefficients["expenv:sex", c(1,2,4)]



  res
})

modcohort <- do.call(rbind,modcohort)



datplot <- data.frame(TE=modcohort[,1], SE=modcohort[,2])

#Perform meta-analysis
metaresTissues <- meta::metagen(TE, SE, data=datplot,
                               studlab= levels(phenored$cohort),
                               level.ci = 0.95, sm="OR")

forest(metaresTissues, layout="JAMA",
       leftlabs=c(" ", "OR (95% CI)"),
       title="", xlab="Sex-E1 interaction risk of obesity")
```

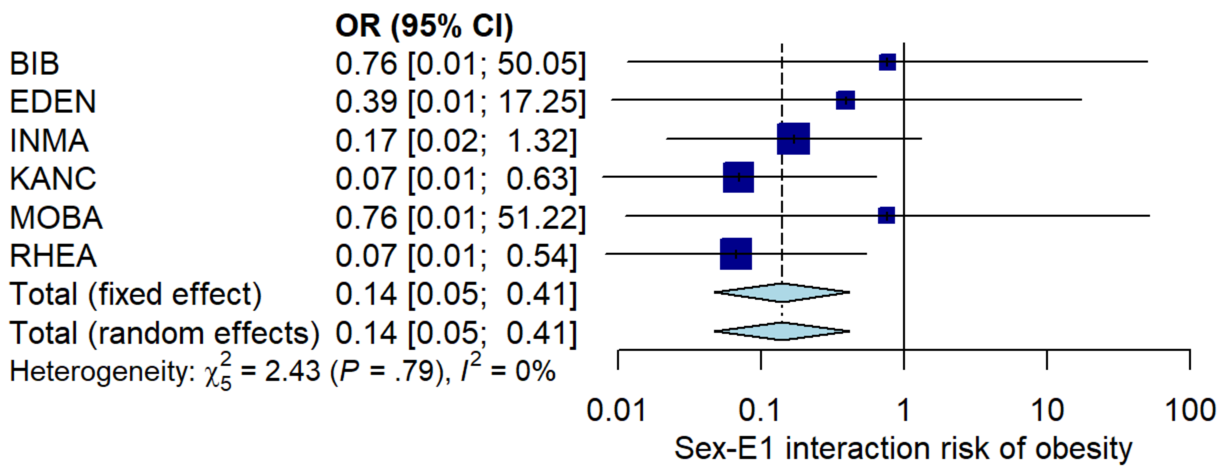

We performed sensitivity analysis, removing the cohorts (BIB and KANC) with the highest levels of imputations for the exposures.

```
### Sensitivity
datplot <- data.frame(TE=modcohort[-c(1,4),1], SE=modcohort[-c(1,4),2])

#Perform meta-analysis
metaresTissues <- meta::metagen(TE, SE, data=datplot,
                             studlab= levels(phenored$cohort)[-c(1,4)],
                             level.ci = 0.95, sm="OR")



metaresTissues
```

```
## Number of studies combined: k = 4
##
##                           OR           95%-CI     z p-value
## Common effect model  0.1504 [0.0409; 0.5530] -2.85  0.0043
## Random effects model 0.1504 [0.0409; 0.5530] -2.85  0.0043
##
## Quantifying heterogeneity:
##  tau^2 = 0 [0.0000; 12.7480]; tau = 0 [0.0000; 3.5704]
##  I^2 = 0.0% [0.0%; 84.7%]; H = 1.00 [1.00; 2.56]
##
## Test of heterogeneity:
##     Q d.f. p-value
##  1.40    3  0.7049
##
## Details on meta-analytical method:
## - Inverse variance method
## - Restricted maximum-likelihood estimator for tau^2
## - Q-profile method for confidence interval of tau^2 and tau
```

```
#stratified
#males
fa <- formula(paste0("y~", paste0(names(phenored)[-c(1,3)],
                                  collapse="+")))

summary(bayesglm(fa, family = "binomial", data = phenored[phenored$sex==0,]))$coefficients["e
xpenv", c(1,4)]
```

```
##   Estimate   Pr(>|z|)
## 1.14423083 0.01204972
```

```
summary(bayesglm(fa, family = "binomial", data = phenored[phenored$sex==1,]))$coefficients["e
xpenv", c(1,4)]
```

```
##      Estimate      Pr(>|z|)
## -1.7077730486  0.0004736414
```

We characterized the environment of protection for females (E1) by its individual associations with each level of the environmental exposures. The environment is characterized by moderate consumption of dairy products and less smoking (protection for girls), green space, and low facility richness (risk for boys).

We tested individual associations between the environment and its constituent exposures.

```
expset <- modexp[,finf]


dims <- lapply(predob$featurenames$featurenames, function(ph)
{
  mod <- "gaussian"

  y <- expset[,ph]
  sex <- phenotype$sex
  cohort <- phenotype$cohort
  covmat <-   phenotype[,covs]
  expenv <- tarob$classification[rownames(phenotype)]


   phenored <- data.frame(y=y,expenv=expenv, covmat, sex=sex)


  fa <- formula(paste0("y~", paste0(names(phenored)[-1],
                                  collapse="+")))



  if(length(table(y))==2){
     mod <- "binomial"
     y <- as.numeric(factor(y))-1
  }

  summary(glm(fa, family = mod, data = phenored))$coefficients["expenv", c(1,4)]
})

dims <- do.call(rbind, dims)
rownames(dims) <-predob$featurenames$featurenames

dims
```

```
##                            Estimate     Pr(>|z|)
## hs_cotinine_mcat_None3   -1.135895 1.257820e-09
## h_dairy_preg_Ter3        -2.381383 8.588392e-43
## h_dairy_preg_Ter2         2.603403 9.025795e-32
## hs_cotinine_mcat_None2   -1.470700 7.133429e-12
## h_frichness300_preg_None -5.897183 5.199473e-34
## h_greenyn300_preg_None1   2.019061 9.713766e-32
```

Sexual dimorphism in obesity and neurodevelopment

We first assessed the association between obesity and the neuropsychological measures, adjusting by covariates

```
#correlation of obesity and other phenotypes, adjusted by covariates

dims <- lapply(nms[-1], function(ph)
{
  y <- phenotype[[ph]]
  obs <- phenotype$hs_bmicat_None
  sex <- as.numeric(phenotype$sex)-1
  cohort <- phenotype$cohort
  covmat <-   phenotype[,covs]


   phenored <- data.frame(y=y, obs=obs, covmat)


  fa <- formula(paste0("y~", paste0(names(phenored)[-1],
                                  collapse="+")))

  summary(glm(fa, family = "binomial", data = phenored))$coefficients["obs", c(1,4)]
})

dims <- do.call(rbind, dims)
rownames(dims) <-nms[-1]

dims[,1] <- exp(dims[,1])
dims
```

```
##                       Estimate   Pr(>|z|)
## hs_correct_raven     2.423287 0.01432970
## hs_accuracy_numeros2 2.659600 0.02851486
## hs_accuracy_ANT      1.488668 0.23348399
## hs_ADHD_raw_Cat      2.157510 0.03292313
```

We tested the association of all clinical outcomes and the sex-environment interaction where boys are likely more obese than girls. We adjusted for all covariates.

```
#sex*dimorphic group

dims <- lapply(nms, function(ph)
{
  mod <- "gaussian"

  y <- phenotype[[ph]]
  expenv <- tarob$classification[rownames(phenotype)]
  sex <- as.numeric(phenotype$sex)-1
  cohort <- phenotype$cohort
  covmat <-   phenotype[,covs]


   phenored <- data.frame(y=y, expenv=expenv, sex=sex, covmat)


  fa <- formula(paste0("y~expenv:sex+", paste0(names(phenored)[-1],
                                collapse="+")))


  if(length(table(y))==2){
     mod <- "binomial"
     y <- as.numeric(factor(y))-1
  }

  summary(glm(fa, family = mod, data = phenored))
})



expenvsexassoc <- t(sapply(dims, function(x) x$coefficients["expenv:sex", c(1,2,4)]))
rownames(expenvsexassoc) <- nms

expenvsexassoc[,c(1,2)] <- exp(expenvsexassoc[,c(1,2)])

expenvsexassoc
```

```
##                         Estimate Std. Error     Pr(>|z|)
## hs_bmicat_None       0.04793845   2.006735 1.291627e-05
## hs_correct_raven     0.42194606   1.544786 4.723905e-02
## hs_accuracy_numeros2 0.31612950   1.644595 2.062344e-02
## hs_accuracy_ANT      1.03036959   1.438712 9.344495e-01
## hs_ADHD_raw_Cat      1.33946651   1.751677 6.021028e-01
```

The complete models, with the covariates’ coefficients, for each neuropsychological measure were

```
names(dims) <- nms
dims[2:5]
```

```
## $hs_correct_raven
##
## Call:
## glm(formula = fa, family = mod, data = phenored)
##
## Deviance Residuals:
##      Min        1Q    Median        3Q       Max
## -1.69161  -0.51612  -0.18498  -0.00011   2.79614
##
## Coefficients:
##                          Estimate Std. Error z value Pr(>|z|)
## (Intercept)            -3.076e+02  4.651e+02  -0.661 0.508380
## expenv                  4.803e-01  2.977e-01   1.614 0.106602
## sex                     2.752e-01  3.622e-01   0.760 0.447345
## cohortEDEN             -1.405e+01  5.319e+02  -0.026 0.978925
## cohortINMA             -5.291e-01  7.127e-01  -0.742 0.457822
## cohortKANC             -1.034e+00  3.380e-01  -3.059 0.002224 **
## cohortMOBA             -1.725e+00  7.652e-01  -2.254 0.024171 *
## cohortRHEA              4.050e-01  3.537e-01   1.145 0.252162
## e3_yearbir_None         1.572e-01  2.312e-01   0.680 0.496560
## h_mbmi_None            -2.782e-02  2.081e-02  -1.337 0.181128
## hs_wgtgain_None         2.564e-02  1.550e-02   1.654 0.098105 .
## e3_gac_None            -3.152e-02  6.755e-02  -0.467 0.640736
## h_age_None             -1.656e-02  2.205e-02  -0.751 0.452633
## h_edumc_None2          -4.806e-01  3.335e-01  -1.441 0.149592
## h_edumc_None3          -9.725e-01  3.347e-01  -2.905 0.003671 **
## h_native_None1         -1.780e+00  1.007e+00  -1.768 0.077127 .
## h_native_None2         -3.955e-01  5.465e-01  -0.724 0.469246
## h_parity_None1          5.554e-01  2.397e-01   2.317 0.020502 *
## h_parity_None2          1.022e+00  2.862e-01   3.571 0.000356 ***
## hs_child_age_days_None -2.410e-03  8.879e-04  -2.714 0.006639 **
## expenv:sex             -8.629e-01  4.349e-01  -1.984 0.047239 *
## ---
## Signif. codes:  0 '***' 0.001 '**' 0.01 '*' 0.05 '.' 0.1 ' ' 1
##
## (Dispersion parameter for binomial family taken to be 1)
##
##     Null deviance: 985.53  on 1038  degrees of freedom
## Residual deviance: 644.81  on 1018  degrees of freedom
##   (5 observations deleted due to missingness)
## AIC: 686.81
##
## Number of Fisher Scoring iterations: 17
##
##
## $hs_accuracy_numeros2
##
## Call:
## glm(formula = fa, family = mod, data = phenored)
##
## Deviance Residuals:
##     Min       1Q   Median       3Q      Max
## -1.3771  -0.4831  -0.3011  -0.2024   3.0036
##
## Coefficients:
```

```
##                         Estimate Std. Error z value Pr(>|z|)
## (Intercept)           -6.081e+02  5.297e+02  -1.148   0.2509
## expenv                 4.626e-01  3.528e-01   1.311   0.1899
## sex                    6.650e-01  3.960e-01   1.679   0.0931 .
## cohortEDEN             2.059e+00  1.363e+00   1.511   0.1309
## cohortINMA             7.852e-01  7.870e-01   0.998   0.3184
## cohortMOBA             1.595e-01  7.430e-01   0.215   0.8300
## cohortRHEA             6.860e-01  4.234e-01   1.620   0.1052
## e3_yearbir_None        3.030e-01  2.632e-01   1.151   0.2496
## h_mbmi_None           -6.298e-03  2.744e-02  -0.230   0.8185
## hs_wgtgain_None       -9.701e-03  1.911e-02  -0.508   0.6118
## e3_gac_None            5.296e-02  8.195e-02   0.646   0.5181
## h_age_None            -2.367e-02  2.695e-02  -0.878   0.3798
## h_edumc_None2          1.574e-01  4.259e-01   0.370   0.7117
## h_edumc_None3          2.375e-01  4.221e-01   0.563   0.5737
## h_native_None1         2.173e+00  1.194e+00   1.820   0.0688 .
## h_native_None2         1.388e+00  1.053e+00   1.318   0.1876
## h_parity_None1         4.742e-01  2.696e-01   1.759   0.0786 .
## h_parity_None2         4.660e-01  3.558e-01   1.310   0.1902
## hs_child_age_days_None -1.987e-03  1.014e-03  -1.960   0.0500 *
## expenv:sex            -1.152e+00  4.975e-01  -2.315   0.0206 *
## ---
## Signif. codes:  0 '***' 0.001 '**' 0.01 '*' 0.05 '.' 0.1 ' ' 1
##
## (Dispersion parameter for binomial family taken to be 1)
##
##     Null deviance: 620.71  on 808  degrees of freedom
## Residual deviance: 499.94  on 789  degrees of freedom
##   (235 observations deleted due to missingness)
## AIC: 539.94
##
## Number of Fisher Scoring iterations: 6
##
##
## $hs_accuracy_ANT
##
## Call:
## glm(formula = fa, family = mod, data = phenored)
##
## Deviance Residuals:
##     Min       1Q   Median       3Q      Max
## -1.5379  -0.6871  -0.4002  -0.1920   2.7954
##
## Coefficients:
##                         Estimate Std. Error z value Pr(>|z|)
## (Intercept)           -5.153e+02  4.098e+02  -1.257  0.20864
## expenv                -3.773e-01  2.345e-01  -1.609  0.10766
## sex                   -6.509e-01  2.878e-01  -2.261  0.02373 *
## cohortEDEN             5.981e-01  1.039e+00   0.575  0.56504
## cohortINMA            -7.039e-02  6.052e-01  -0.116  0.90741
## cohortKANC             3.647e-02  3.262e-01   0.112  0.91099
## cohortMOBA             6.256e-01  5.398e-01   1.159  0.24652
## cohortRHEA             6.264e-01  3.521e-01   1.779  0.07519 .
## e3_yearbir_None        2.581e-01  2.036e-01   1.267  0.20500
## h_mbmi_None            4.248e-02  1.805e-02   2.354  0.01859 *
## hs_wgtgain_None        1.585e-02  1.352e-02   1.172  0.24127
```

```
## e3_gac_None            -2.861e-02  5.638e-02  -0.507  0.61189
## h_age_None             -2.645e-02  2.034e-02  -1.300  0.19349
## h_edumc_None2          -1.793e-02  3.223e-01  -0.056  0.95562
## h_edumc_None3          -3.196e-01  3.246e-01  -0.985  0.32481
## h_native_None1         -5.687e-01  9.268e-01  -0.614  0.53952
## h_native_None2          3.364e-01  4.436e-01   0.758  0.44822
## h_parity_None1          5.545e-01  2.041e-01   2.717  0.00659 **
## h_parity_None2          3.886e-01  2.549e-01   1.525  0.12727
## hs_child_age_days_None -1.303e-03  7.233e-04  -1.801  0.07174 .
## expenv:sex              2.992e-02  3.637e-01   0.082  0.93445
## ---
## Signif. codes:  0 '***' 0.001 '**' 0.01 '*' 0.05 '.' 0.1 ' ' 1
##
## (Dispersion parameter for binomial family taken to be 1)
##
##     Null deviance: 1030.38  on 1028  degrees of freedom
## Residual deviance:  856.47  on 1008  degrees of freedom
##   (15 observations deleted due to missingness)
## AIC: 898.47
##
## Number of Fisher Scoring iterations: 6
##
##
## $hs_ADHD_raw_Cat
##
## Call:
## glm(formula = fa, family = mod, data = phenored)
##
## Deviance Residuals:
##     Min       1Q   Median       3Q      Max
## -1.0313  -0.5129  -0.3574  -0.2355   2.8390
##
## Coefficients:
##                          Estimate Std. Error z value Pr(>|z|)
## (Intercept)            834.998352 532.694850   1.567  0.11700
## expenv                   0.199198   0.285936   0.697  0.48602
## sex                     -1.219917   0.487135  -2.504  0.01227 *
## cohortEDEN               1.811851   1.142373   1.586  0.11273
## cohortINMA               1.313139   0.698598   1.880  0.06015 .
## cohortKANC               0.510177   0.447952   1.139  0.25474
## cohortMOBA               0.137559   0.723829   0.190  0.84928
## cohortRHEA               0.013304   0.502850   0.026  0.97889
## e3_yearbir_None         -0.414731   0.264635  -1.567  0.11707
## h_mbmi_None             -0.001170   0.023255  -0.050  0.95987
## hs_wgtgain_None          0.014792   0.017245   0.858  0.39103
## e3_gac_None              0.042572   0.074251   0.573  0.56641
## h_age_None              -0.017844   0.025461  -0.701  0.48339
## h_edumc_None2            0.222393   0.358693   0.620  0.53525
## h_edumc_None3           -0.428176   0.373932  -1.145  0.25218
## h_native_None1           0.456677   1.020489   0.448  0.65451
## h_native_None2           0.308389   0.647637   0.476  0.63395
## h_parity_None1          -0.025741   0.243780  -0.106  0.91591
## h_parity_None2          -0.811019   0.396722  -2.044  0.04092 *
## hs_child_age_days_None  -0.002418   0.000874  -2.767  0.00566 **
## expenv:sex               0.292271   0.560574   0.521  0.60210
## ---
```

```
## Signif. codes:  0 '***' 0.001 '**' 0.01 '*' 0.05 '.' 0.1 ' ' 1
##
## (Dispersion parameter for binomial family taken to be 1)
##
##     Null deviance: 675.54  on 1036  degrees of freedom
## Residual deviance: 610.70  on 1016  degrees of freedom
##   (7 observations deleted due to missingness)
## AIC: 652.7
##
## Number of Fisher Scoring iterations: 6
```

Methylomic associations for exposure environment of obesity dimorphism

We asked whether the classification of individuals into the environment of female protection of obesity against obesity (E1) was associated with molecular data. We performed differential methylation analysis for the environment, using `limma` , adjusting for the covariates previously used in the causal modeling. We additionally adjusted by inferred immune cell count in blood.

```
##methylation

load("./data/Methy_final.RData")

methy <- methy_final
genesIDsmet <-  rowData(methy)

phenotype_expression <-  Biobase::pData(methy)

comnames <- intersect(rownames(phenotype), rownames(phenotype_expression))


covimmune <- phenotype_expression[comnames,c("NK_6", "Bcell_6","CD4T_6","CD8T_6","Gran_6","Mo
no_6")]


eff <- phenotype[comnames,]
phenotype_expression <- phenotype_expression[comnames, ]
covmat <- eff[comnames, covs]

phenored <- data.frame(ex=tarob$classification[comnames],
                       t=data4teffob$teffdata[comnames,"t"],
                       covmat, covimmune)


fa <- formula(paste0("~", paste0(names(phenored),
                                  collapse="+")))


mod <- model.matrix(fa, data = phenored)

cnm <- intersect(colnames(methy), rownames(mod))

met <- getBeta(methy[, cnm])

lmfit <- lmFit(met, design = mod[cnm,])
lmFite <- eBayes(lmfit)
tab <- topTable(lmFite, n = Inf, coef = "ex")

resmet <- data.frame(tab[,c(1,4,5)], gene=genesIDsmet[rownames(tab),"UCSC_RefGene_Name"])

head(resmet, 4)
```

	logFC <dbl>	P.Value <dbl>	adj.P.Val <dbl>	gene <chr>
cg02876062	-0.009988068	2.367265e-06	0.6065047	FAM107B
cg04752591	0.011901210	3.264876e-06	0.6065047	ZBTB9
cg21285773	-0.005499663	9.121493e-06	0.9998542	CYMP
cg19182289	0.008337350	2.697458e-05	0.9998542	RGL2

4 rows

We performed enrichment analysis using the `enrichGO` function of clusterProfiler.

```
load("./data/Trans_final.Rdata")
genexpr <- trans_final
genesIDs <-  Biobase::fData(genexpr)

tmet <- topTable(lmFite, coef="ex", number=Inf)
selgenes <- rownames(tmet)[tmet$P.Value<0.01]
selgenes <- unique(genesIDsmet[selgenes, "UCSC_RefGene_Name"])
selgenes <- genesIDs[genesIDs[,"GeneSymbol_Affy"]%in%selgenes, "EntrezeGeneID_Affy"]
selgenes <- selgenes[!selgenes%in%c("NA", "")]
selgenes <- sapply(strsplit(selgenes, ";"), function(x) x[[1]])

#run enrichment in GO
GOmet1 <- enrichGO(gene = selgenes, 'org.Hs.eg.db', ont="BP", pvalueCutoff=0.01, pAdjustMetho
d="fdr")

clusterProfiler::dotplot(GOmet1)
```

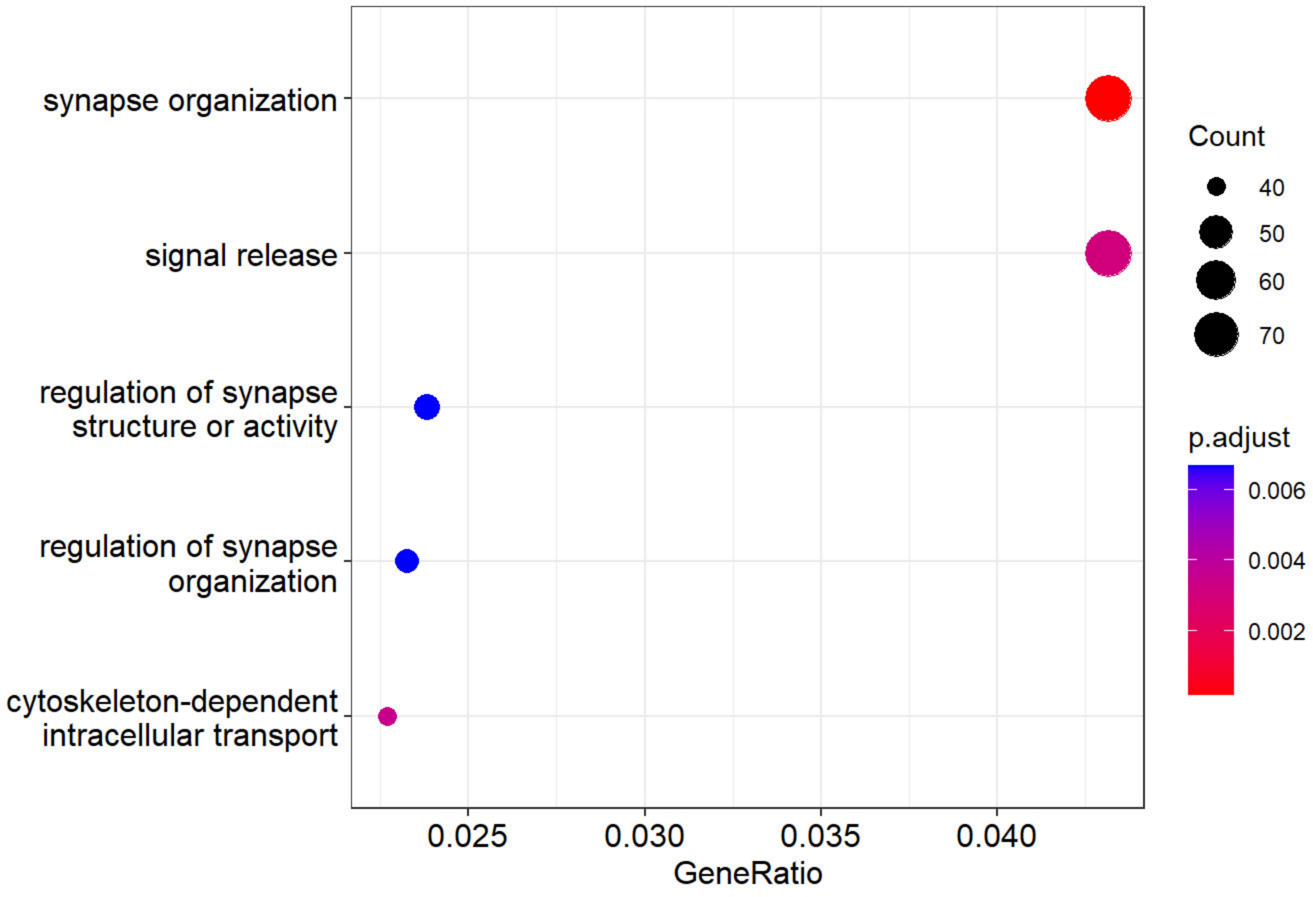


```
GOmet2 <- enrichGO(gene = selgenes, 'org.Hs.eg.db', ont="MF", pvalueCutoff=0.01, pAdjustMetho
d="fdr")

clusterProfiler::dotplot(GOmet2)
```

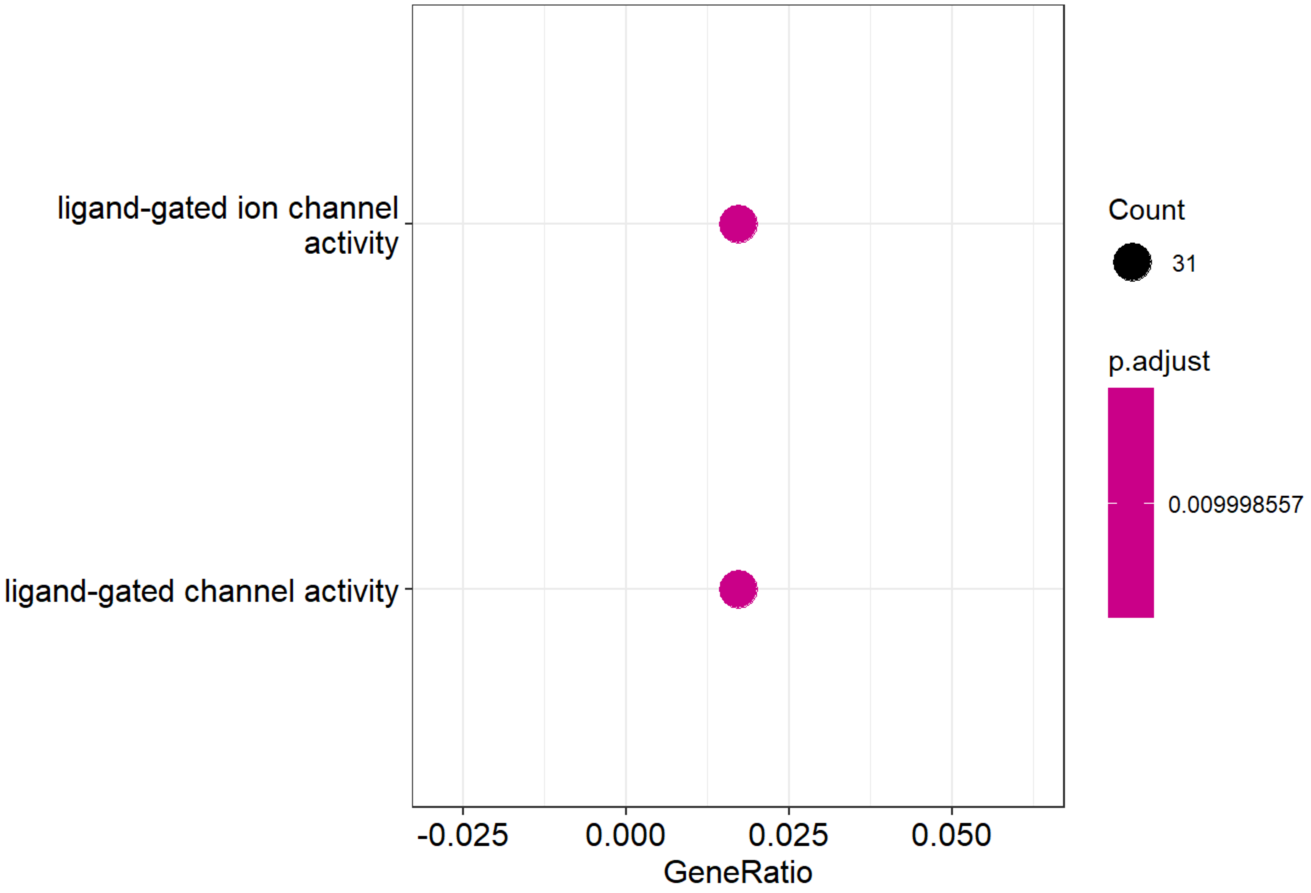


```
GOmet3 <- enrichGO(gene = selgenes, 'org.Hs.eg.db', ont="CC", pvalueCutoff=0.01, pAdjustMethod="fdr")

clusterProfiler::dotplot(GOmet3)
```

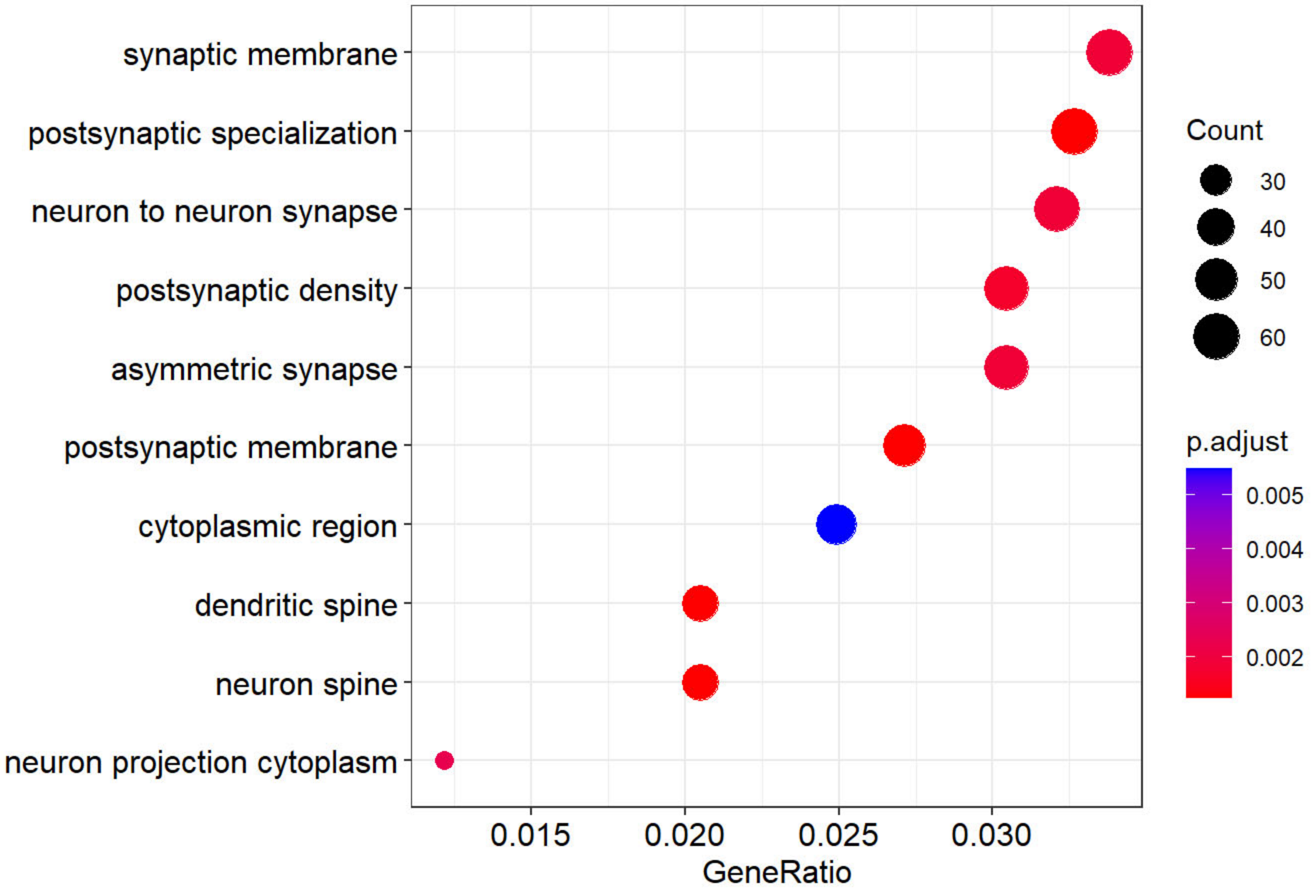
synaptic membrane
postsynaptic specialization
neuron to neuron synapse
postsynaptic density
asymmetric synapse
postsynaptic membrane
cytoplasmic region
dendritic spine
neuron spine
neuron projection cytoplasm
0.015
0.020
0.025
0.030
GeneRatio
Count
30
40
50
60
p.adjust
0.005
0.004
0.003
0.002
